# Genome-wide identification of transcriptional targets of RORA reveals direct regulation of multiple genes associated with autism spectrum disorder

**DOI:** 10.1186/2040-2392-4-14

**Published:** 2013-05-22

**Authors:** Tewarit Sarachana, Valerie W Hu

**Affiliations:** 1 Department of Biochemistry and Molecular Medicine, The George Washington University School of Medicine and Health Sciences, 2300 I Street NW, Washington, DC, 20037, USA; 2 Current address: Center for Biologics Evaluation and Research, Food and Drug Administration, 8800 Rockville Pike, Bethesda, MD, 20892, USA

**Keywords:** RORA, Autism, Nuclear hormone receptor, Transcriptional targets, Chromatin immunoprecipitation, Promoter microarray

## Abstract

**Background:**

We have recently identified the nuclear hormone receptor *RORA* (retinoic acid-related orphan receptor-alpha) as a novel candidate gene for autism spectrum disorder (ASD). Our independent cohort studies have consistently demonstrated the reduction of *RORA* transcript and/or protein levels in blood-derived lymphoblasts as well as in the postmortem prefrontal cortex and cerebellum of individuals with ASD. Moreover, we have also shown that *RORA* has the potential to be under negative and positive regulation by androgen and estrogen, respectively, suggesting the possibility that *RORA* may contribute to the male bias of ASD. However, little is known about transcriptional targets of this nuclear receptor, particularly in humans.

**Methods:**

Here we identify transcriptional targets of *RORA* in human neuronal cells on a genome-wide level using chromatin immunoprecipitation (ChIP) with an anti-RORA antibody followed by whole-genome promoter array (chip) analysis. Selected potential targets of *RORA* were then validated by an independent ChIP followed by quantitative PCR analysis. To further demonstrate that reduced *RORA* expression results in reduced transcription of *RORA* targets, we determined the expression levels of the selected transcriptional targets in *RORA*-deficient human neuronal cells, as well as in postmortem brain tissues from individuals with ASD who exhibit reduced *RORA* expression.

**Results:**

The ChIP-on-chip analysis reveals that RORA1, a major isoform of RORA protein in human brain, can be recruited to as many as 2,764 genomic locations corresponding to promoter regions of 2,544 genes across the human genome. Gene ontology analysis of this dataset of genes that are potentially directly regulated by RORA1 reveals statistically significant enrichment in biological functions negatively impacted in individuals with ASD, including neuronal differentiation, adhesion and survival, synaptogenesis, synaptic transmission and plasticity, and axonogenesis, as well as higher level functions such as development of the cortex and cerebellum, cognition, memory, and spatial learning. Independent ChIP-quantitative PCR analyses confirm binding of RORA1 to promoter regions of selected ASD-associated genes, including *A2BP1, CYP19A1, ITPR1, NLGN1,* and *NTRK2*, whose expression levels (in addition to *HSD17B10*) are also decreased in *RORA1*-repressed human neuronal cells and in prefrontal cortex tissues from individuals with ASD.

**Conclusions:**

Findings from this study indicate that RORA transcriptionally regulates *A2BP1, CYP19A1, HSD17B10, ITPR1, NLGN1,* and *NTRK2*, and strongly suggest that reduction of this sex hormone-sensitive nuclear receptor in the brain causes dysregulated expression of these ASD-relevant genes as well as their associated pathways and functions which, in turn, may contribute to the underlying pathobiology of ASD.

## Background

Autism spectrum disorder (ASD) is a neurodevelopmental disorder that is characterized by deficits in social understanding and interactions, aberrant communication, and repetitive, stereotyped behaviors, often with restricted interests [1-4]. With an overall prevalence of 1 in 88 individuals in the United States [5], autism is inexplicably biased towards males by a ratio of at least 4:1, although some recent studies [6,7] have reported ratios closer to 2:1, depending on the population studied. The consistently observed male bias, however, has prompted theories that elevated fetal or neonatal testosterone levels may be a risk factor for ASD [8], and the recent association of various autism traits in individuals who presented prenatally with elevated testosterone levels in amniotic fluid has supported this hypothesis [9,10]. However, the molecular and physiological mechanism(s) that lead to elevated testosterone levels in individuals with ASD, both prenatally and postnatally, remain essentially unknown.

We have recently identified a novel autism candidate gene, retinoic acid-related (RAR) orphan receptor-alpha (*RORA*) [11] which is regulated by male and female hormones in a manner that may provide an explanation for the higher testosterone levels and, possibly, sex bias in ASD [12]. RORA is a ligand-dependent orphan nuclear hormone receptor that, in combination with co-regulator proteins, serves as a transcriptional regulator. Although RORA has never before been associated with ASD, our recent studies have demonstrated: reduced expression of *RORA* in lymphoblastoid cell lines (LCL) derived from individuals with autism [13]; increased methylation leading to reduced expression of *RORA* in the LCL from cases vs. sibling controls [11]; and decreased expression of RORA protein in the prefrontal cortex and the cerebellum of individuals with autism [11]. Together, these results link these molecular changes in RORA in blood-derived peripheral cells to molecular pathology in the brain tissues of individuals with autism.

These findings are notable because studies on the Rora-deficient *staggerer* mouse model indicate that Rora is involved in several processes potentially relevant to autism, including Purkinje cell differentiation [14,15], cerebellar development [16,17], protection of neurons against oxidative stress [18], suppression of inflammation [19], and regulation of circadian rhythm [20]. Indeed, the involvement of Purkinje cells and cerebellar abnormalities as well as neuroinflammation and oxidative stress in the autistic brain has been comprehensively discussed in a consensus report on the pathological role of the cerebellum in autism [21]. Recently, the proposed circadian dysfunction in ASD [22,23] has also been supported by both genetic studies that have identified polymorphisms in “clock” (circadian regulator) genes [24] as well as gene expression analyses that identified *RORA* as one of the 15 differentially expressed circadian genes in a phenotypic subgroup of individuals with ASD with severe language impairment [13]. The known functions of *Rora* in the mouse model thus appear to be relevant to the observed pathological findings in humans with ASD. Moreover, behavioral studies on the *staggerer* mouse, primarily used as a model to study ataxia and dystonia [16], show that RORA is also associated with restricted behaviors reminiscent of autism, such as perseverative tendencies [25], limited maze patrolling [26], anomalous object exploration [27], and deficits in spatial learning [28]. Although there are currently no reported studies on the social behaviors of *staggerer* mice, it is clear that RORA is associated with at least some of the symptomatology and pathology of autism.

As a transcriptional regulator, RORA is known to bind DNA as a monomer or as a homodimer to hormone response elements upstream of target genes to modulate expression of those genes. In *staggerer* mice that exhibit spontaneous disruption of the *Rora* gene, Rora deficiency has been reported to cause aberrant expression of several genes involved in Purkinje cell differentiation (for example, *Shh*), calcium-mediated signaling (for example, *Itpr1, Calb1, Cals1, Pcp4*), as well as excitatory neurotransmission at glutamatergic synapses of Purkinje cells in the cerebellum (for example, *Slc1a6, Grm1, Pcp2*) [29]. In mouse skeletal muscle, Rora influences genes associated with lipid and carbohydrate metabolism (for example, *Acsl4, Cd36, Hif1a*), LXR nuclear receptor signaling (for example, *LXRa, Srebp-1c*), and Akt and AMPK signaling (for example, *Akt2*) [30]. Liver tissues of Rora-deficient mice have been shown to exhibit altered expression of several genes involved in triglyceride synthesis and storage (for example, *Cidec, Cidea, Mogat1*) [31], thus demonstrating pleiotropic effects of *Rora* depending on tissue type. However, little is known about transcriptional targets of RORA in humans, particularly in the central nervous system. We therefore sought to identify, at the genome-wide level, putative transcriptional targets of RORA in human neuronal cells, and to validate a functionally relevant subset of targets that may play a role in ASD. Since we have previously demonstrated decreased *RORA* expression in the frontal cortex of individuals with autism relative to that of unaffected controls [11,12], we also investigated mRNA expression of the confirmed RORA targets in postmortem brain tissues of individuals diagnosed with autism in comparison with the expression of those genes in the brain of sex-matched and age-matched unaffected individuals.

## Methods

### Cell culture

The human neuroblastoma cells SH-SY5Y (ATCC, Manassas,VA, USA) were cultured in 1:1 Modified Eagle’s Medium (MEM) and Ham’s F12 media (MediaTech, Manassas, VA, USA) supplemented with 15% (v/v) fetal bovine serum (Atlanta Biologicals, Lawrenceville, GA, USA) and 1% penicillin/streptomycin (MediaTech). Cells were maintained at 37°C with 5% CO_2_, and split 1:2 every 3 or 4 days when the cells reached ~80% confluency. For harvesting, the cells were treated with trypsin-ethylenediamine tetraacetic acid (MediaTech) for 2 to 3 minutes to release them from the surface of the culture flask. Complete growth medium was then added to the flask containing suspension cells to inactivate trypsin. Cells were transferred to a sterile centrifuge tube and pelleted by spinning at 800 rpm for 5 minutes at 4°C and gently washed twice with ice-cold PBS.

### Frozen human brain tissues

Frozen postmortem prefrontal cortex (BA9/10) tissues from male individuals with autism (*n* = 3) and from age-matched typically developing males (*n* = 3) were obtained through the Autism Tissue Program (San Diego, CA, USA). All frozen brain tissues were preserved by the Harvard Brain Tissue Resource Center, Harvard Medical School. The list of human brain tissues used in this study is shown in Additional file 1, along with information on the age of the donor, the postmortem interval (PMI) between death and tissue collection, and the cause of death when known.

### Chromatin immunoprecipitation (ChIP)-on-chip analysis

Chromatin immunoprecipitation (ChIP) for promoter array analysis was performed using the Millipore EZ-ChIP Chromatin Immunoprecipitation Kit (Millipore, Billerica, MA, USA) according to the manufacturer's protocol. Briefly, the human neuronal cell line SH-SY5Y was cultured in complete growth medium in a T-175 flask until ~80% confluency (approximately 1.5 × 10^7^ cells). The medium was then carefully removed without disturbing the cells. The cells were fixed with 37% formaldehyde for exactly 10 minutes to crosslink chromatin. The crosslinking reaction was terminated by addition of 10% glycine, after which ice-cold PBS supplemented with protease inhibitor cocktail was added to the culture flask to wash and chill the cells. The crosslinked cells were dislodged from the flask by scraping gently with a cell scraper and then transferred to a pre-chilled centrifuge tube. Crosslinked cells were pelleted and nuclear extraction was performed using the Active Motif Nuclear Extract Kit (Active Motif, Carlsbad, CA, USA) according to the manufacturer's protocol. Nuclear pellets were resuspended in SDS Lysis Buffer and sonicated on wet ice to shear crosslinked chromatin to 200–1,000 bp using a Heat Systems-Ultrasonics W-380 sonicator (Heat Systems-Ultrasonics/Misonix, Farmingdale, NY, USA) set to 30% of maximum output power for 20 × 10 seconds with 1-minute intervals. Sonicated chromatin was divided into several aliquots for immunoprecipitation reactions and stored at −80°C until use. Each immunoprecipitation reaction was conducted using 1 μg of goat anti-RORA1 or normal goat IgG antibody (Santa Cruz Biotechnology, Santa Cruz, CA, USA). Immunoprecipitated chromatin was then reverse-crosslinked by adding 5 M NaCl to the final concentration of 0.2 M and incubated at 65°C overnight. DNA from the immunoprecipitated chromatin was isolated and purified using Millipore DNA purification columns (Millipore) and then submitted to the Genomics Core Facility at The George Washington University for analysis on Affymetrix human promoter tiling arrays (Affymetrix, Santa Clara, CA, USA).

### Analysis of RORA binding sites on Affymetrix promoter tiling arrays

Purified DNA from the chromatin immunoprecipitated with anti-RORA1 or control IgG antibodies was amplified using the Whole Genome Amplification 2 Kit (Sigma Aldrich, St. Louis, MO, USA) according to the manufacturer’s protocol. The amplification product of each immunoprecipitation reaction was purified and hybridized on Affymetrix GeneChip Human Promoter 1.0R microarrays. Probes significantly enriched in RORA1-immunoprecipitated DNA relative to IgG-immunoprecipitated DNA were identified using the Partek Genomics Suite software (Partek, St. Louis, MO, USA), as described in detail below.

The Affymetrix Human Promoter 1.0R array contains over 4.6 × 10^6^ probes tiled over 25,500 promoter regions of annotated genes. Each gene promoter is thus interrogated on average by over 150 probes or “tiles”. For this study, DNA immunoprecipitated from SH-SY5Y cells with either a RORA1-specific antibody or a non-specific IgG as a control was hybridized on separate chips in triplicate, and the amount of probe hybridized to each element on the chips was determined. Partek Genomics Suite Software was used to analyze the intensities of the probe elements across the promoter array using the workflow recommended by Affymetrix for tiling arrays. In brief, the data normalization procedures include adjusting for probe sequence, RMA background correction, quantile normalization, and log (base 2) transformation of intensity data. Two-way analysis of variance was then used to determine differences between the RORA1-co-immunoprecipitated DNA and IgG-co-immunoprecipitated control DNA in hybridizing to specific probes. The MAT (model-based analysis of tiling arrays) peak-seeking algorithm [32] was subsequently used to detect enriched regions in the RORA1-co-immunoprecipated DNA versus IgG control samples. Nominal *P* value cutoff ≤0.05 (corresponding to false discovery rate (FDR) <7%), MAT score >0, minimum number of probes for each region >10, and average length for the ChIP fragment >600 bp were used as the peak detection parameters in this discovery phase of high-throughput screening for potential RORA targets. The microarray data from this study have been deposited into the Gene Expression Omnibus repository [GEO: GSE45756]. Positive MAT scores for a specific region indicate that RORA binding is enriched in that region relative to IgG controls. The genes mapping to the RORA-enriched promoter regions were identified using the gene annotation database (hg18) provided by Affymetrix. To further aid in the selection of gene promoters for confirmation analyses, an additional level of analysis was performed to determine the average intensity of probes across the regions hybridizing to the RORA-immunoprecipitated DNA relative to that hybridizing to the control DNA. These intensities were used to calculate the fold-change or enrichment across each gene promoter region for binding of the RORA-immunoprecipitated DNA over that of the IgG-precipitated DNA. Among the RORA-enriched promoter sites, the site corresponding to *ITPR1* was used as a positive control since *Itpr1* had been identified as a transcriptional target of Rora in mice [29]. Because the *ITPR1* promoter region exhibited a fold-change of 1.29, we used this value as a minimum average fold-change in selecting target genes for further confirmation.

### Rationale for selection of putative target genes for further confirmation

Of the 2,544 genes identified as putative targets of RORA by the ChIP-on-chip analysis (see Results), we selected *ITPR1, CYP19A1, HSD17B10, A2BP1 (RBFOX1), NLGN1,* and *NTRK2* for further confirmation by ChIP-quanititative PCR analyses and by functional knockdown of *RORA*. Among these putative target genes, *A2BP1, ITPR1, NLGN1*, and *NTRK2* have been previously identified as candidate genes for ASD [33-35]. The protein product of *CYP19A1*, also known as aromatase, is involved in the enzymatic conversion of androgen to estrogen via testosterone intermediates. Although *CYP19A1* had previously been reported to be a target of RORA in human breast cancer cell lines [36], we recently demonstrated that its promoter region was a site of RORA binding in a human neuronal cell line [12]. Here, we wished to further confirm *CYP19A1* as a transcriptional target of RORA by functional knockdown studies. Like *CYP19A1*, *HSD17B10* also codes for an enzyme involved in the conversion of androgen to estrogen, but via 19-hydroxyandrostenedione, 19-oxyandrostenedione and estrone intermediates. Because of our earlier study demonstrating significant and correlated reductions in RORA and CYP19A1 protein levels in the autistic brain [12], we were interested in determining whether a deficiency in RORA could also impact this alternate biochemical pathway through down-regulation of *HSD17B10*, thus potentially reinforcing the buildup of androgen and reduction of estrogen in neuronal cells and tissues. The relevance of these genes to ASD is further elaborated in the Discussion.

### Prediction of RORA binding elements

Putative binding elements of RORA in the promoter regions of *A2BP1, CYP19A1, HSD17B10, ITPR1, NLGN1*, and *NTRK2* were predicted using PROMO 3.0 [37,38], JASPAR [39], and the EpiTect ChIP Search Portal of SABiosciences (Valencia, CA, USA). For each putative target gene, a total of three or four predicted transcription factor binding sites within 10 kb upstream of the transcription start site were selected for ChIP-quantitative PCR analyses.

### ChIP-quantitative PCR analysis

Chromatin immunoprecipitation for ChIP-quantitative PCR analysis of RORA binding elements in the promoter region of putative RORA targets was conducted using the ChIP-IT Express Enzymatic Kit (Active Motif) following the manufacturer’s instructions. Briefly, confluent SH-SY5Y cells (approximately 1.5 × 10^7^ cells in a T-175 flask) were fixed with 10% formaldehyde for exactly 10 minutes and the fixation reaction was stopped by adding 10% glycine to the flask. The cells were washed with 10 ml ice-cold PBS for 5 seconds, then 6 ml ice-cold PBS supplemented with 0.5 mM (final concentration) phenylmethylsulfonylfluoride supplied in the kit was added to the culture flask to wash and chill the cells. The crosslinked cells were transferred from the flask to a pre-chilled 15 ml centrifuge tube by scraping gently with a cell scraper. Crosslinked cells were pelleted by centrifugation for 10 minutes at 2,500 rpm (720 relative centrifugal force) at 4°C, and then resuspended in 1 ml ice-cold Active Motif Lysis Buffer (Active Motif). The crosslinked cells were transferred to an ice-cold dounce homogenizer and the nuclei were released from the cells by douncing with a tight pestle. Optimal cell lysis was assessed under a phase contrast microscope using a hemacytometer. The nuclei were then transferred to an ice-cold 1.7 ml microcentrifuge tube and pelleted by centrifugation for 10 minutes at 5,000 rpm (2,400 relative centrifugal force) at 4°C. Chromatin was then isolated from the nuclear pellets and sheared into 150 to 1,000 bp fragments by incubating with 10 U/ml (final concentration) Enzymatic Shearing Cocktail (Active Motif) at 37°C for exactly 10 minutes. The enzymatic shearing reaction was stopped by adding ethylenediamine tetraacetic acid to a final concentration of 10 mM and chilling the reaction tube on ice for 10 minutes. Optimal shearing was assessed by agarose gel electrophoresis. For each ChIP reaction, enzymatically-sheared chromatin containing ~7 to 25 μg chromatin DNA was immunoprecipitated using 1 μg antibody and 25 μl Protein G Magnetic Beads (Active Motif). The list of antibodies for ChIP analyses is shown in Additional file 2. Immunoprecipitated chromatin was reverse-crosslinked according to the ChIP-IT Express Enzymatic Kit protocol and DNA was isolated and purified from the chromatin using the ChIP DNA Purification Kit (Active Motif).

Real-time quantitative PCR analysis was conducted using the Applied Biosystems 7300 Real-Time PCR System (Applied Biosystems, Foster City, CA, USA) to determine the enrichment of each RORA binding element in immunoprecipitated DNA. Primers for quantitative PCR analysis were designed using Primer3 software [40] and were synthesized by Integrated DNA Technologies (Coralville, IA, USA). Input DNA was diluted into five 10-fold serial dilutions and included in quantitative PCR analyses. Relative enrichment values of RORA binding elements in each immunoprecipitated chromatin were calculated using standard curves obtained from the enrichment of RORA binding elements in the 10-fold serial dilutions of respective input DNA. The list of primers is shown in Additional file 3.

### Short hairpin RNA (shRNA) transfection

SH-SY5Y cells were cultured in a six-well culture plate containing complete growth medium without antibiotics (approximately 2.5 × 10^5^ cells per well) until cells were ~70% confluent. For each well, the cells were transfected with 2.50 μg *RORA1* shRNA (Santa Cruz Biotechnology) or 2.50 μg negative control shRNA (Santa Cruz Biotechnology) using Lipofectamine LTX and PLUS reagent (Invitrogen, Carlsbad, CA, USA) according to the manufacturer's protocol. Briefly, shRNA (2.50 μg) was diluted in 500 μl Opti-MEM I Reduced Serum Medium (Invitrogen) and 1.25 μl PLUS reagent was added to the diluted shRNA solution. Lipofectamine LTX (25 μl) was then added to the shRNA-PLUS solution, incubated for 25 minutes at room temperature to form shRNA-Lipofectamine-LTX-PLUS complexes, and added to the cells. The cells were then incubated with the shRNA-Lipofectamine-LTX-PLUS complexes at 37°C and 5% CO_2_ for 24 hours before harvesting. The list of shRNAs is shown in Additional file 2.

### RNA isolation and quantitative RT-PCR analysis

Quantitative RT-PCR analyses were performed as previously described [41]. Total RNA from the shRNA-transfected cells was isolated using TRIzol (Invitrogen) and purified using the RNeasy Mini Kit (Qiagen, Gaithersburg, MD, USA) following the manufacturers' instructions. Human brain tissues were homogenized in the Bullet Blender Homogenizer (Next Advance, Averill Park, NY, USA) using nuclease-free glass beads, after which total RNA from homogenized brain tissues was isolated using the RNeasy Mini Kit (Qiagen). A total of 1 μg purified total RNA was used for cDNA synthesis using the iScript cDNA Synthesis Kit (BioRad, Hercules, CA, USA) according to the manufacturer’s protocols. The reaction (20 μl) was incubated at 25°C for 5 minutes, followed by 42°C for 30 minutes, and ending with 85°C for 5 minutes. After reverse transcription, the cDNA reaction mixture was diluted to a volume of 50 μl with nuclease-free water and used as a template for quantitative PCR analyses. Real-time PCR analyses were conducted using the Applied Biosystems 7300 Real-Time PCR System (Applied Biosystems). Primers for quantitative RT-PCR analyses designed by Primer3 software are listed in Additional file 3. The relative quantity of transcripts in each sample was calculated using standard curves based on the relative quantity of 18S RNA transcript in 10-fold serial dilutions of the respective sample.

### Statistical analysis

A paired, two-sided, Student’s *t* test was performed to determine significance of the differences in numerical data obtained by quantitative PCR analyses. *P* <0.05 was considered statistically significant.

Hypergeometric distribution analyses [42] were used to determine the statistical significance of enrichment in autism candidate genes among the transcriptional targets identified by ChIP-on-chip analysis, relative to the genes present in AutDB [34], AutismKB [35], or a combination of the two databases of autism-associated genes.

### Pathway and gene ontology analyses

Network prediction, functional, and gene ontology analyses were accomplished using licensed Ingenuity Pathway Analysis (IPA) and Pathway Studio7.0 software, as well as the open-access DAVID Bioinformatics Resources 6.7 [43,44]. For functional and network analyses, Fisher exact *P* values were calculated using the entire set of genes in the Ingenuity Knowledge Base as the reference set.

## Results

### Identification of RORA transcriptional targets using whole-genome promoter array analysis and gene ontology analyses

ChIP-on-chip analysis revealed that a total of 2,764 probes (corresponding to 2,544 unique genes) were significantly enriched in RORA-immunoprecipitated chromatin relative to IgG-immunoprecipitated chromatin *(P* <0.05; FDR <7%). The complete list of gene-associated regions from the ChIP-on-chip analyses is shown in Additional file 4. Gene ontology analysis of this complete list of genes using DAVID Bioinformatics Resources 6.7 shows that neuron differentiation and development as well as axonogenesis are significantly over-represented among biological processes shown in Annotation cluster 1 which has a highly significant Enrichment Score (ES) of 5.107 (Table 1). The dataset is also significantly enriched for genes involved in synaptic transmission and plasticity (Annotation cluster 4 with enrichment score 3.456) and post-synaptic density (Annotation cluster 5 with enrichment score 3.176). Furthermore, it is remarkable that *all* of genes in annotation clusters 1 to 5 are contained in AutDB and/or AutismKB databases. (See Additional file 5 for a full list of genes and functions associated with annotation clusters 2 to 5.) This is noteworthy because, although this nuclear hormone receptor is known to have pleiotropic functions in different tissues, neurological functions are clearly enriched among the putative transcriptional targets of RORA within the context of a human neuronal cell line.

**Table 1:** Top annotation cluster from gene ontology analysis of 2,544 potential transcriptional targets of RORA

**Annotation cluster 1: enrichment score 5.107**				
**GO term**	**Count**	***P*Value**	Benjamini*	**Genes**
GO:0030182-neuron differentiation	35	1.52E-08	3.56E-05	RAB3A, CDK5R1, ADORA2A, PAX3, RORA, RTN1, EPHB2, ARX, ATP2B2, BDNF, SLC1A3, LAMB2, CD44, CXCR4, ANK3, DMD, ROBO3, LHX8, LMX1B, MDGA2, PTPRR, NTNG1, RPGRIP1, NUMBL, SOD2, PTPN11, CTNNA2, SLITRK1, NTRK1, NTRK2, FOXG1, CNTN4, CACNA1F, CUX1, NTM
GO:0048666-neuron development	28	2.96E-07	3.48E-04	RAB3A, CDK5R1, ADORA2A, EPHB2, ARX, ATP2B2, BDNF, SLC1A3, LAMB2, CD44, ANK3, CXCR4, DMD, ROBO3, LHX8, LMX1B, NTNG1, RPGRIP1, CTNNA2, PTPN11, SOD2, NUMBL, SLITRK1, FOXG1, NTRK2, CNTN4, CACNA1F, NTM
GO:0000904-cell morphogenesis involved in differentiation	22	1.86E-06	1.45E-03	RAB3A, CDK5R1, NTNG1, PTPN11, CTNNA2, EPHB2, NUMBL, ARX, SLITRK1, ATP2B2, DAB2, BDNF, SLC1A3, LAMB2, CXCR4, ANK3, LAMA5, FOXG1, CNTN4, ROBO3, CACNA1F, FN1
GO:0000902-cell morphogenesis	27	2.53E-06	1.48E-03	RAB3A, CDK5R1, ADORA2A, EPHB2, ARX, ATP2B2, BDNF, DAB2, SLC1A3, LAMB2, ANK3, CXCR4, DMD, MKKS, ROBO3, FN1, NTNG1, MARK2, CTNNA2, PTPN11, NUMBL, SLITRK1, LAMA5, FOXG1, CNTN4, CACNA1F, CDC42BPB
GO:0032989-cellular component morphogenesis	28	6.15E-06	2.88E-03	RAB3A, CDK5R1, ADORA2A, EPHB2, ARX, ATP2B2, DAB2, BDNF, SLC1A3, LAMB2, ANK3, CXCR4, DMD, OBSL1, MKKS, ROBO3, FN1, NTNG1, MARK2, CTNNA2, PTPN11, NUMBL, SLITRK1, LAMA5, FOXG1, CNTN4, CACNA1F, CDC42BPB
GO:0048858-cell projection morphogenesis	21	7.30E-06	2.14E-03	RAB3A, CDK5R1, ADORA2A, NTNG1, PTPN11, CTNNA2, EPHB2, NUMBL, ARX, SLITRK1, BDNF, LAMB2, CXCR4, ANK3, LAMA5, DMD, FOXG1, MKKS, CNTN4, ROBO3, CACNA1F
GO:0048667-cell morphogenesis involved in neuron differentiation	19	1.01E-05	2.63E-03	RAB3A, CDK5R1, NTNG1, PTPN11, CTNNA2, EPHB2, NUMBL, ARX, SLITRK1, ATP2B2, BDNF, SLC1A3, LAMB2, CXCR4, ANK3, FOXG1, CNTN4, ROBO3, CACNA1F
GO:0048812-neuron projection morphogenesis	19	1.31E-05	3.07E-03	RAB3A, CDK5R1, ADORA2A, NTNG1, PTPN11, CTNNA2, EPHB2, NUMBL, ARX, SLITRK1, BDNF, LAMB2, CXCR4, ANK3, DMD, FOXG1, CNTN4, ROBO3, CACNA1F
GO:0030030-cell projection organization	26	1.39E-05	2.96E-03	MTSS1, RAB3A, CDK5R1, DNAH9, ADORA2A, EPHB2, ARX, ATP2B2, BDNF, LAMB2, CD44, ANK3, CXCR4, DMD, MKKS, ROBO3, FGD3, NTNG1, CTNNA2, PTPN11, NUMBL, SLITRK1, LAMA5, FOXG1, CNTN4, CACNA1F
GO:0032990-cell part morphogenesis	21	1.40E-05	2.73E-03	RAB3A, CDK5R1, ADORA2A, NTNG1, PTPN11, CTNNA2, EPHB2, NUMBL, ARX, SLITRK1, BDNF, LAMB2, CXCR4, ANK3, LAMA5, DMD, FOXG1, MKKS, CNTN4, ROBO3, CACNA1F
GO:0031175-neuron projection development	20	4.65E-05	6.80E-03	RAB3A, CDK5R1, ADORA2A, NTNG1, PTPN11, CTNNA2, EPHB2, NUMBL, ARX, SLITRK1, BDNF, LAMB2, CD44, CXCR4, ANK3, DMD, FOXG1, CNTN4, ROBO3, CACNA1F
GO:0007409-axonogenesis	17	4.98E-05	6.86E-03	RAB3A, CDK5R1, NTNG1, PTPN11, CTNNA2, EPHB2, NUMBL, ARX, SLITRK1, BDNF, LAMB2, CXCR4, ANK3, FOXG1, CNTN4, ROBO3, CACNA1F
GO:0007411-axon guidance	9	7.12E-03	2.67E-01	ARX, CDK5R1, BDNF, CXCR4, ANK3, FOXG1, CNTN4, ROBO3, EPHB2

Results were obtained using DAVID Bioinformatics Resources 6.7. The enrichment score is the negative logarithm of the average of *P* values for items within each cluster. *P values corrected for multiple testing.

To further mine the dataset of potential transcriptional targets for higher level biological functions, disorders, and canonical pathways, we conducted network prediction and functional analysis on the RORA-bound genes using Ingenuity Pathway Analysis (IPA) software. Table 2 summarizes the functional and pathway analysis which reveals that nervous system development and function, neurological disease, and axonal guidance signaling are among the top five most significantly over-represented biological functions, disorders, and canonical pathways, respectively, associated with the RORA-enriched gene dataset. More detailed investigation into the specific neurological functions associated with the genes showed significant enrichment of genes involved in development of the brain and nervous system, axonogenesis, cell-cell adhesion of neurons, long-term potentiation of granule cells, neuritogenesis, and development of the cerebellum (Table 3). The genes associated with neurological disorders/diseases were enriched for schizophrenia, Huntington’s disease, movement disorders, dyskinesia, and seizure disorder, the latter three of which often present with the most severe subtype of ASD, which we have found to be associated with RORA deficiency [13]. Interestingly, a number of behaviors that are often disrupted or impaired in ASD, such as cognition, learning, circling behavior, emotional behavior, memory and spatial learning, are also significantly over-represented in the dataset of putative transcriptional targets of RORA. The complete list of genes associated with each of the biological functions, diseases/disorders, and behaviors described in Table 3 are provided in Additional file 6.

**Table 2:** Top five biological functions, disorders, and canonical pathways associated with 2,544 potential transcriptional targets of RORA

**Biological functions, disorders, and pathways**	***P*value**	**Number of molecules**
**Physiological system development and function**		
Organismal survival	3.56E-08 to 6.82E-04	305
Nervous system development and function	5.83E-08 to 4.28E-03	344
Tissue morphology	5.83E-08 to 4.72E-03	419
Cardiovascular system development and function	8.89E-08 to 4.72E-03	268
Embryonic development	9.01E-08 to 4.72E-03	370
**Diseases and disorders**		
Hereditary disorder	8.09E-06 to 1.56E-03	204
Neurological disease	8.09E-06 to 4.72E-03	292
Psychological disorders	8.09E-06 to 8.09E-06	107
Cancer	1.65E-05 to 4.77E-03	731
Hematological disease	1.65E-05 to 4.67E-03	166
**Top canonical pathways**		**Ratio**
Superpathway of inositol phosphate compounds	4.97E-04	37/227
Production of nitric oxide and reactive oxygen species in macrophages	1.29E-03	35/210
Axonal guidance signaling	2.47E-03	69/468
3-phosphoinositide biosynthesis	2.52E-03	29/175
Small cell lung cancer signaling	2.60E-03	17/89

These data were obtained using Ingenuity Pathway Analysis software. *P* values were calculated using Fisher's exact test, which was performed using the entire set of genes within the Ingenuity Knowledge Base as the reference set.

**Table 3:** Top neurological diseases, disorders, and behaviors associated with the 2,544 potential transcriptional targets of RORA

**Function annotation (number of molecules)**	***P*value**
**Nervous system development and function**	
Quantity of neurons (67)	5.83E-08
Development of brain (102)	9.01E-08
Morphology of nervous system (161)	3.96E-07
Morphology of nervous tissue (118)	5.44E-07
Development of central nervous system (122)	1.50E-06
Axonogenesis (43)	1.68E-05
Morphology of nerves (30)	7.32E-05
Cell-cell adhesion of neurons (6)	1.48E-04
Morphology of central nervous system (98)	1.90E-04
Morphology of rhombencephalon (35)	2.41E-04
Quantity of sensory neurons (19)	2.81E-04
Development of nerves (19)	2.81E-04
Abnormal morphology of neurons (51)	2.85E-04
Morphology of neurons (55)	3.37E-04
Antinociception (13)	3.84E-04
Development of neurons (37)	3.98E-04
Morphology of brain (88)	4.40E-04
Development of interneurons (7)	4.56E-04
Migration of neurons (46)	4.79E-04
Abnormal morphology of cerebellum (21)	5.58E-04
Development of metencephalon (24)	5.88E-04
Loss of neurons (29)	8.01E-04
Long-term potentiation of granule cells (4)	8.18E-04
Abnormal morphology of cranial nerve (19)	8.60E-04
Neuritogenesis (83)	1.11E-03
Development of cerebellum (23)	1.22E-03
Development of forebrain (45)	1.26E-03
Fasciculation of nervous tissue (9)	1.34E-03
Abnormal morphology of granule cells (12)	1.42E-03
Development of globus pallidus (3)	1.56E-03
Activation of hippocampus (3)	1.56E-03
Abnormal morphology of hair cells (9)	1.84E-03
Morphogenesis of neurites (58)	1.85E-03
Adhesion of neuronal cells (14)	1.88E-03
Abnormal morphology of outer hair cells (6)	2.01E-03
Sensory system development (23)	2.09E-03
Development of rhombencephalon (28)	2.15E-03
Chemotaxis of granule cells (4)	2.23E-03
Guidance of axons (31)	2.24E-03
Morphology of mechanosensory neurons (10)	2.49E-03
Cell viability of neurons (39)	2.51E-03
Analgesia (18)	2.62E-03
Synaptic transmission of cerebral cortex cells (11)	3.03E-03
Memory (39)	3.52E-03
Neurotransmission (71)	3.75E-03
Development of cranial nerve (11)	3.79E-03
Cell viability of granule cells (8)	4.28E-03
**Neurological disease**	
Schizophrenia (107)	8.09E-06
Chorea (117)	3.23E-04
Huntington's Disease (116)	4.09E-04
Movement Disorders (183)	9.11E-04
Dyskinesia (118)	1.27E-03
Neurological signs (120)	1.45E-03
Familial transthyretin amyloidosis (3)	1.56E-03
Seizure disorder (60)	3.92E-03
Disorder of basal ganglia (135)	4.31E-03
Polymicrogyria (4)	4.72E-03
**Behavior**	
Behavior (180)	2.74E-07
Cognition (79)	1.70E-05
Learning (74)	3.41E-05
Circling behavior (12)	1.11E-03
Emotional behavior (42)	3.04E-03
Memory (39)	3.52E-03
Spatial learning (33)	4.23E-03

These data were obtained using Ingenuity Pathway Analysis software. *P* values were calculated using Fisher's exact test, which was performed using the entire set of genes within the Ingenuity Knowledge Base as the reference set.

To determine whether the ChIP-on-chip-identified putative targets of RORA were enriched in autism candidate genes, hypergeometric distribution analyses were performed to calculate *P* values for over-representation of genes from two autism gene databases (AutDB and AutismKB) within the target gene list. The results of the hypergeometric distribution analyses (Table 4) indicate that autism candidate genes, identified through various other studies, are indeed enriched among our transcriptional targets.

**Table 4:** Hypergeometric distribution analysis results to determine enrichment of autism candidate genes from AutDB and AutismKB

**Autism gene database**	**Total number of genes in database**	**Total promoter regions on array**	**Total IP RORA-binding promoter regions**	**RORA-binding autism genes (overlap)**	***P*value**
AutDB (SFARI gene)	328	25,500	2,544	49	0.0028
AutismKB (syndromic + non-syndromic)	3,050	25,500	2,544	426	<0.001
AutDB + AutismKB	3,158	25,500	2,544	438	<0.001

For the hypergeometric distribution analyses, the total number of general markers (that is, the population) is the number of genes (25,500) whose promoter regions are represented on the array, and the total number of selected markers is the number of unique genes (2,544) identified as targets of RORA by the ChIP-on-chip analysis. The total interesting markers are the number of autism candidate genes in the respective or combined databases, and the number of selected interesting markers are the genes within our dataset that overlap with those in either or the combined databases.

### Network prediction of selected transcriptional targets to assess relevance to autism spectrum disorder (ASD)

**Figure 1:**
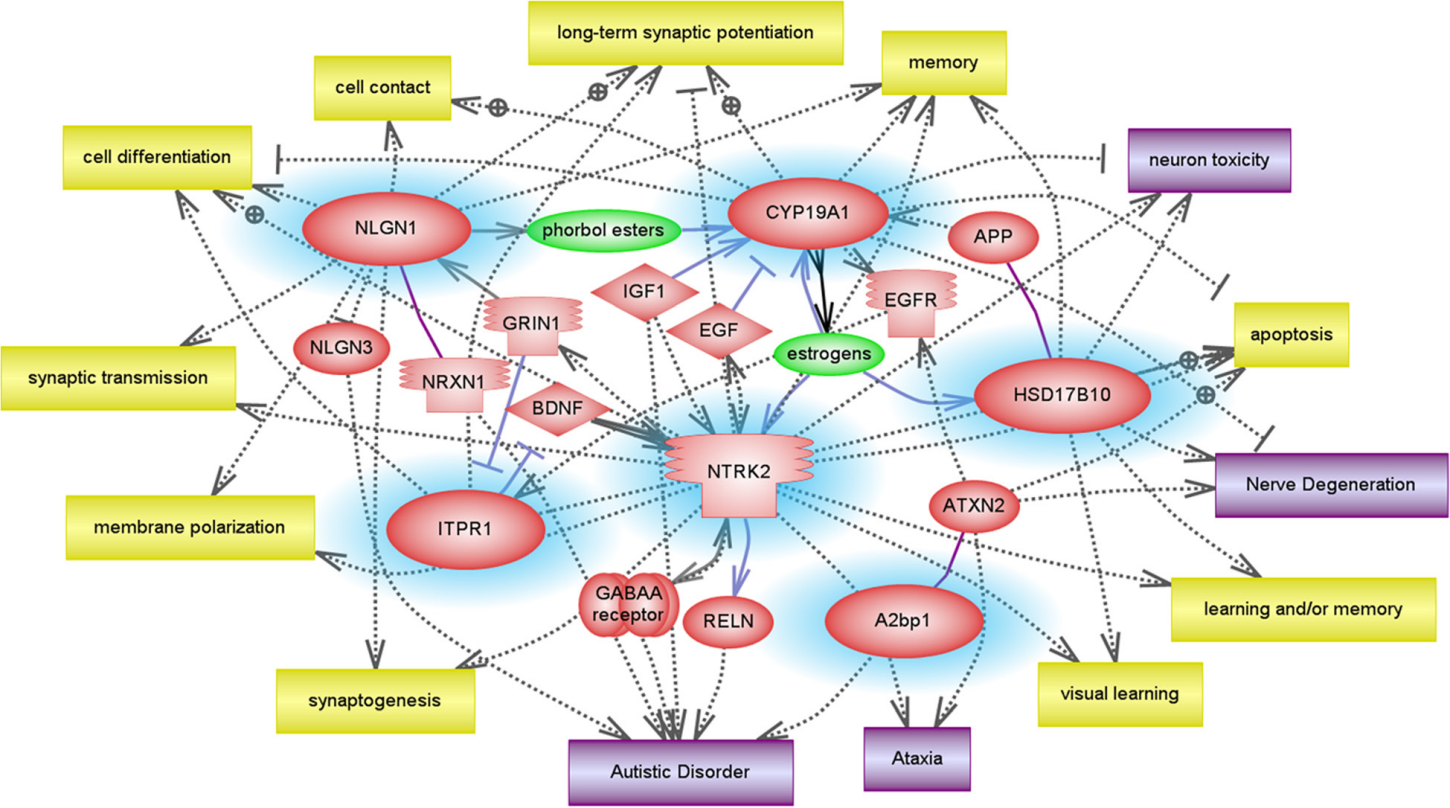
**Pathway analysis of selected potential transcriptional targets of RORA.** A biological network was created using the Pathway Studio 7.0 program to identify biological functions and disorders associated with potential RORA transcriptional targets selected for confirmation (that is, *ITPR1*, *CYP19A1*, *A2BP1*, *HSD17B10*, *NLGN1*, and *NTRK2* which are highlighted with a blue halo).

We selected six potential gene targets, *ITPR1*, *CYP19A1*, *A2BP1*, *HSD17B10*, *NLGN1*, and *NTRK2,* for confirmation by independent ChIP-quantitative PCR and functional analyses based on the reasons given in Methods. The probe enrichment data and genomic locations for these genes are shown in Table 5. Functional analysis of these six genes using the Pathway Studio 7.0 network prediction program revealed an association of these genes with neurological disorders, including autism and ataxia, as well as autism-related neurological functions, including synaptogenesis, synaptic transmission, long-term potentiation, learning, and memory (Figure 1). Interestingly, other autism candidate genes, including *NLGN3*, *NRXN1*, *RELN*, and *GABAA,* were also included in the predicted gene network, thus supporting the investigation of our selected genes as ASD-relevant transcriptional targets of RORA.

**Table 5:** Enrichment data and genomic location of the RORA-binding enriched regions closest to the selected genes

**Nearest gene**	**Entrez gene name**	**MAT score on T (R vs. G)**	***P*value (region)**	**FDR (%)**	**Average intensity fold-change (R vs. G)**	**Chromosome (strand)**	**Region start**	**Length (bps)**	**Number of probes in region**
**ITPR1**	Inositol 1,4,5-triphosphate receptor, type 1	17.19	0.005	<5	1.29	chr3 (+)	4,724,792	2,804	71
**HSD17B10**	Hydroxysteroid (17-β) dehydrogenase 10	14.11	0.007	<5	11.86	chrX (−)	53,483,92	1,723	42
**A2BP1 (RBFOX1)**	RNA binding protein, fox-1 homolog	12.86	0.009	<5	1.29	chr16 (+)	7,315,418	2,636	68
**NLGN1**	Neuroligin 1	7.30	0.036	<5	3.27	chr3 (+)	174,777,672	639	18
**CYP19A1 (aromatase)**	Cytochrome P450, family 19, subfamily A, polypeptide 1	7.02	0.040	<5	1.61	chr15 (−)	49,415,636	2,111	54
**NTRK2**	Neurotrophic tyrosine kinase, receptor, type 2	6.72	0.044	<7	2.17	chr9 (+)	86,412,498	966	21

*FDR* false discovery rate, *G* intensity of probes hybridized with DNA fragments immunoprecipitated with nonspecific IgG, *MAT* model-based analysis of tiling arrays, *R* intensity of probes hybridized with DNA fragments immunoprecipitated with anti-RORA antibody, *T t*-statistic.

### Confirmation of RORA binding to selected transcriptional targets in human neuronal cells

**Figure 2:**
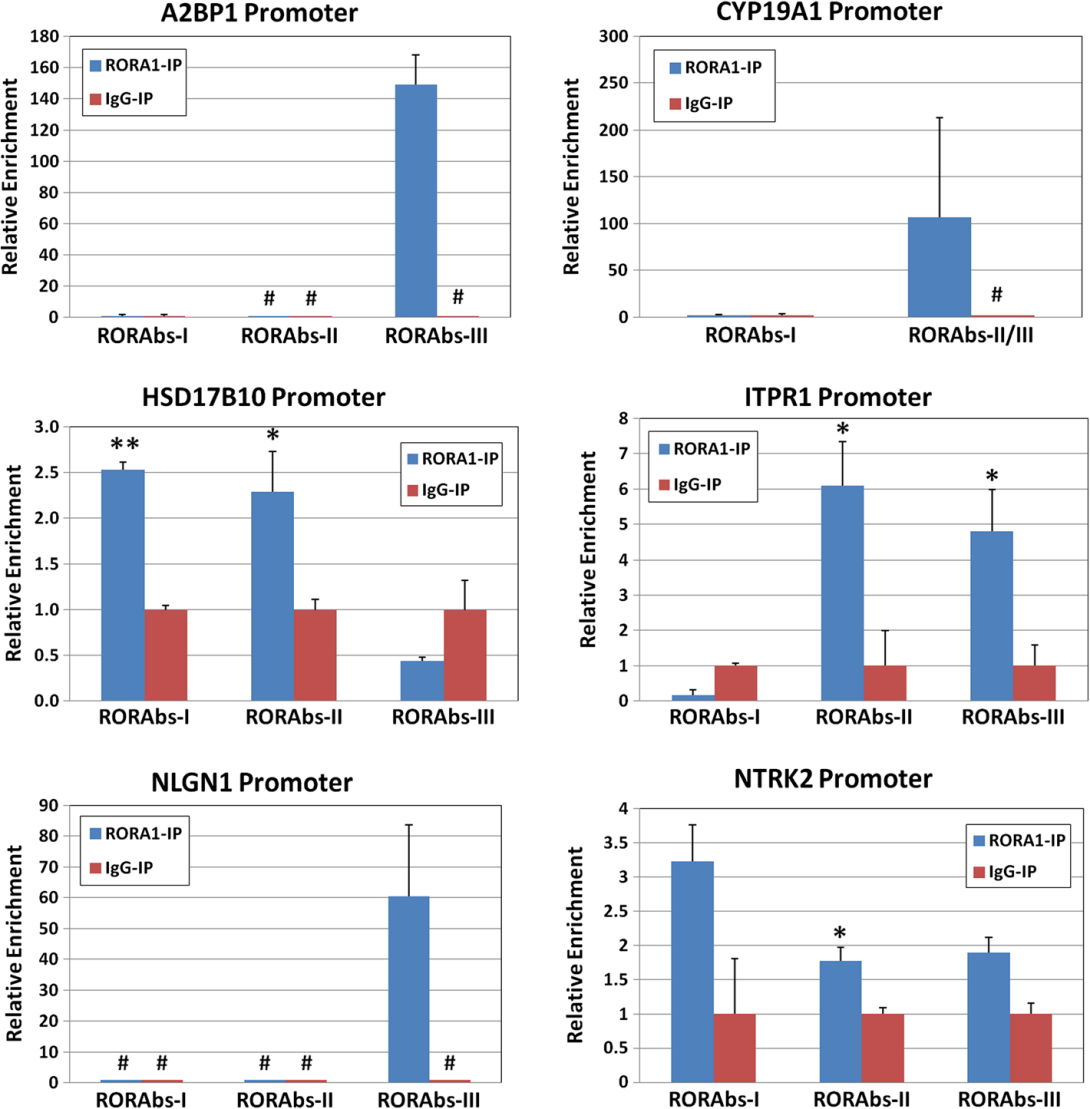
**Chromatin immunoprecipitation-quantitative PCR of RORA transcriptional targets.** Chromatin immunoprecipitation followed by quantitative PCR analysis was conducted to determine whether RORA protein binds to *A2BP1*, *CYP19A1*, *HSD17B10*, *ITPR1*, *NLGN1*, and *NTRK2* promoters. Chromatin was isolated from SH-SY5Y cells and immunoprecipitated with anti-RORA1 or IgG antibody. DNA was purified from the immunoprecipitated chromatin and quantitative real-time PCR analysis (*n* = 3) was performed to determine the enrichment of each RORA binding element in the promoter region of the selected potential RORA targets. Error bars indicate standard error of the mean. * *P* <0.05; ** *P* <0.01; ^#^undetectable.

To confirm that RORA protein binds to the promoter region of these six potential transcriptional targets, we conducted ChIP using anti-RORA1 or nonspecific IgG antibody, followed by quantitative PCR analysis to determine the enrichment of RORA binding sites in the respective promoter regions of the selected genes. Figure 2 shows that RORA binding sites in the promoter region(s) of each of these potential targets were significantly enriched in chromatin samples immunoprecipitated by anti-RORA1 antibody relative to IgG-immunoprecipitated chromatin, indicating that RORA protein indeed binds to promoter regions of *A2BP1*, *CYP19A1*, *HSD17B10*, *ITPR1*, *NLGN1*, and *NTRK2*.

### *RORA* suppression reduces expression of selected transcriptional targets in human neuronal cells

**Figure 3:**
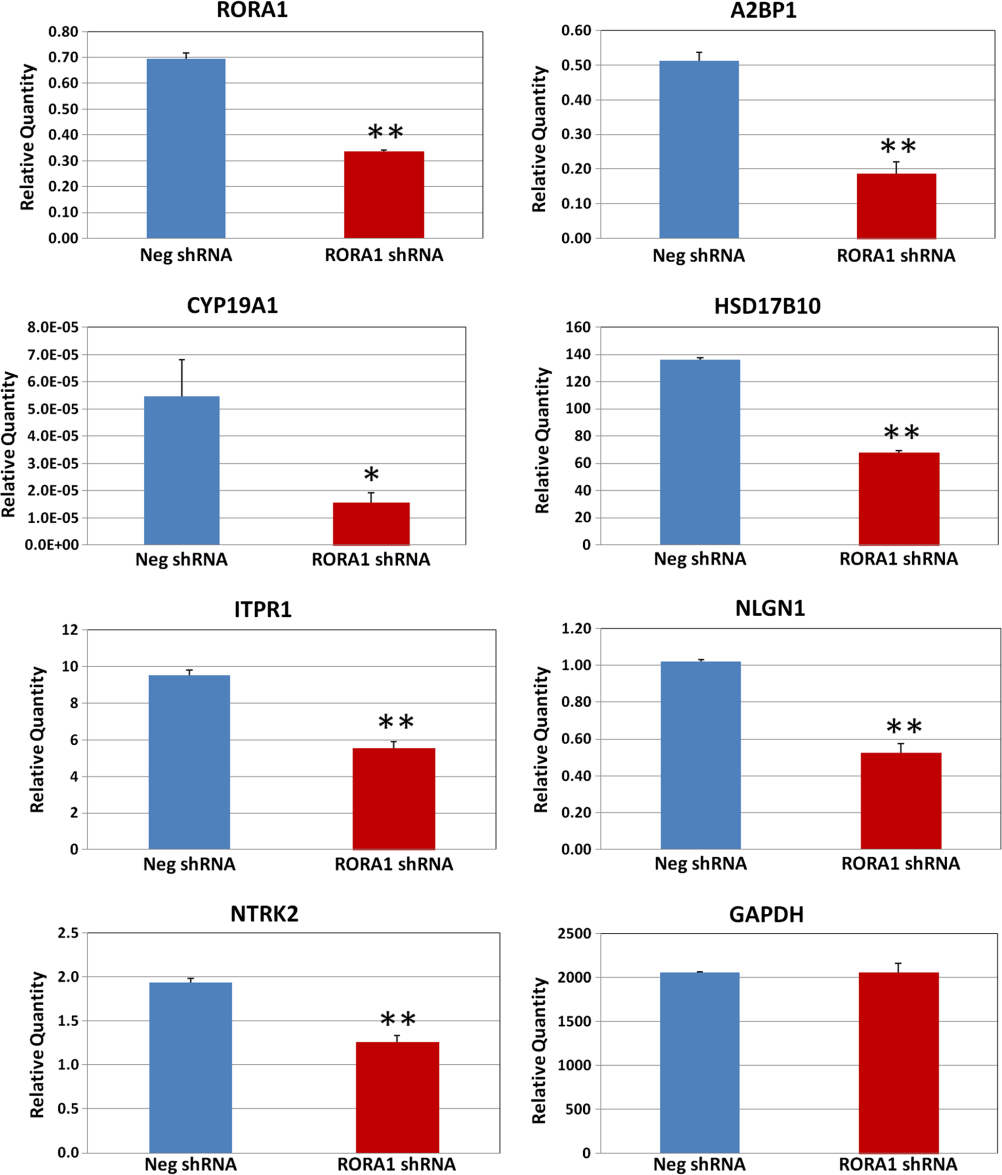
**Quantitative RT-PCR analysis of RORA short hairpin RNA-transfected SH-SY5Y cells.** SH-SY5Y cells were transfected with RORA1 short hairpin RNA (shRNA) or negative control shRNA for 24 hours and quantitative RT-PCR analysis (*n* = 3) was performed to determine expression of *RORA1* and potential transcriptional targets in the transfected cells. *GAPDH* was included as a negative control. Error bars indicate standard error of the mean. *P <0.05, **P <0.01.

To further validate that RORA regulates expression of *A2BP1*, *CYP19A1*, *HSD17B10*, *ITPR1*, *NLGN1*, and *NTRK2*, and to examine whether reduction of *RORA* expression leads to reduced expression of these selected RORA transcriptional targets, shRNA-mediated knockdown of RORA was performed in SH-SY5Y cells using RORA1 shRNA, and quantitative RT-PCR analyses were conducted to measure expression of *RORA1* and the potential targets. Expression of *RORA1* in SH-SY5Y cells was significantly down-regulated by approximately 50% in comparison with cells transfected with negative control shRNA, indicating that shRNA-mediated knockdown of RORA1 was successful. Notably, expression of all selected RORA transcriptional targets was significantly reduced (Figure 3), indicating that expression of these genes is regulated by RORA and that these genes indeed are RORA transcriptional targets in human neuronal cells. As a negative control, the expression level of *GAPDH*, which is not a transcriptional target of RORA, is unaffected by transfection of the SH-SY5Y cells with RORA1 shRNA.

### Expression of transcriptional targets of RORA is relatively reduced in the frontal cortex of individuals with autism in comparison with age-matched controls

We have previously reported reduction of RORA transcript and/or protein in four independent cohorts using LCL as well as tissues from the prefrontal cortex and the cerebellum of individuals diagnosed with autism [11-13]. To examine whether RORA reduction in the brain of individuals with autism may be associated with aberrant expression of the transcriptional targets identified in this study, a pilot study involving quantitative RT-PCR analysis of frozen postmortem prefrontal cortex tissues from individuals with autism (*n* = 3) and age-matched controls (*n* = 3) was performed. As shown in Table 6, the average expression of each of the six gene targets in the combined cases is reduced relative to the average expression levels of the respective genes in the combined controls, although the *P* values (all *P* >0.05) indicate that the differences are not statistically significant.

**Table 6:** Quantitative RT-PCR analysis of post-mortem brain tissues from individuals with autism

**Sample comparison (A vs. C)**	**Age (A, C)**	**RORA**			**CYP19A1**			**ITPR1**			**A2BP1**		
		**Control**	**Autistic**	**A/C**	**Control**	**Autistic**	**A/C**	**Control**	**Autistic**	**A/C**	**Control**	**Autistic**	**A/C**
5144A vs. 5251C	20, 19	6.4	2.0	0.316	1.7E-04	5.2E-05	0.307	127.7	70.8	0.554	553.1	105.3	0.190
5173A vs. 5873C	30, 28	38.4	25.4	0.661	1.2E-02	5.9E-03	0.487	246.4	27.3	0.111	830.1	74.2	0.089
6337A vs. 5718C	22, 22	7.9	0.4	0.046	7.7E-06	1.0E-07	0.013	217.4	176.3	0.811	246.0	64.6	0.263
Average ratio (A/C)		17.6	9.3	**0.527**	4.1E-03	2.0E-03	**0.484**	197.2	91.5	**0.464**	543.1	81.4	**0.150**
*P* value (unpaired *t* test)				0.562			0.660			0.137			0.052
		**HSD17B10**			**NLGN1**			**NTRK2**					
		**Control**	**Autistic**	**A/C**	**Control**	**Autistic**	**A/C**	**Control**	**Autistic**	**A/C**			
5144A vs. 5251C	20, 19	128.7	15.7	0.122	178.3	23.8	0.133	3218.9	544.6	0.169			
5173A vs. 5873C	30, 28	126.3	41.1	0.325	255.3	44.9	0.176	2600.1	1077.2	0.414			
6337A vs. 5718C	22, 22	47.6	39.2	0.824	84.8	61.5	0.725	929.8	1265.1	1.361			
Average ratio (A/C)		100.9	32.0	**0.317**	172.8	43.4	**0.251**	2249.6	962.3	**0.428**			
*P* value (unpaired *t* test)				0.069			0.062			0.147			

Gene expression in brain tissues from individuals with autism (indicated by ’A’) was compared with that in controls (indicated by ’C’). The numbers preceding ‘A’ or ‘C’ identify specific individuals whose brains were donated to the Autism Tissue Program (San Diego, CA, USA). Total RNA was isolated and purified from frozen post-mortem prefrontal cortex tissues from individuals with autism (*n* = 3) and the controls (*n* = 3). Quantitative RT-PCR analyses (in triplicate) were performed to measure expression of *RORA* and that of six transcriptional targets. The transcript levels of each gene were calculated using standard curves obtained from relative *18S* expression in 10-fold serial dilutions of each sample. The numbers highlighted in bold-face type indicate down-regulation of gene expression in autism brain tissues relative to that in respective matched controls. The *P* values are from unpaired *t* tests of the expression data from the combined cases versus that of the combined controls.

## Discussion

Based on reduced expression of *RORA* in LCL and in postmortem brain tissues of individuals with ASD versus unaffected controls, coupled with its known functions in cerebellar development and neuroprotection against inflammation and oxidative stress in mice, we postulated that this nuclear hormone receptor may be responsible for at least some of the pathobiology associated with ASD. In particular, our recent finding that RORA specifically binds the promoter region of aromatase, whose protein expression in the human brain is highly correlated with that of RORA, suggests a molecular explanation for the increased levels of testosterone observed in some individuals with ASD [12]. Specifically, we anticipate that downregulation of aromatase would lead to a buildup of its substrate, testosterone, with a corresponding reduction in estrogen synthesis. Moreover, the downregulation of *RORA* expression in response to androgen and upregulation in response to estrogen suggest a mechanism for the introduction of sex bias in ASD.

As a nuclear hormone receptor, RORA, in combination with various co-regulator proteins, can potentially regulate the transcription of a large number of gene targets. This study was therefore conducted in order to identify additional transcriptional targets of RORA, specifically within the context of a human neuronal cell model. To our knowledge, this is the first ChIP-on-chip study directed towards the comprehensive identification of transcriptional targets of RORA at the genome-wide level in any species. Consequently, in this pilot study aimed at discovery of genes that may be regulated by RORA, we chose to use less stringent *P* values (*P* ≤0.05; FDR <7%) for identification of RORA-enriched regions in order to capture as many potential gene targets of RORA as possible. Not surprisingly, our ChIP-on-chip analysis identified 2,764 promoter regions enriched for RORA binding sites, which corresponded to 2,544 unique genes. Interestingly, gene ontology analysis of this complete set of putative target genes revealed a strikingly high enrichment in genes associated with neuron differentiation and development, neuron projection morphogenesis, axonogenesis, and axon guidance in the top functional annotation cluster (enrichment score 5.107). Intriguingly, *all* of the genes associated with these processes are listed in the AutDB (SFARI Gene) and/or AutismKB databases of autism-associated genes, indicating relevance of our target gene set to ASD. Two additional highly significant functional annotation categories of genes are related to synaptic transmission and plasticity (enrichment score 3.456) and postsynaptic density (enrichment score 3.176). Again, all of the genes in both annotation categories are contained within one of the two aforementioned databases. The statistically significant enrichment of autism candidate genes within our dataset of genes identified by ChIP-on-chip analysis was also confirmed by hypergeometric distribution analyses which used the number of genes in either or both of the autism databases as the total number of interesting markers and the overlap between the genes in our dataset (that is, the selected markers) and the genes in either database as the number of selected interesting markers. The total number of annotated genes on the microarray represents the total number of general markers (that is, the population).

Biological network and pathway analysis using Ingenuity Pathway Analysis software further revealed that these potential transcriptional targets of RORA are significantly associated with nervous system development and function including development of cortex and cerebellum, axonogenesis, adhesion of neuronal cells, neuronal migration, neuritogenesis, neurotransmission, and synaptic density, all of which have been associated with autism [45-50]. It is also noteworthy that neurological disorders known to be co-morbid with autism, including schizophrenia [51,52], movement disorder [53-57], and seizure disorder [58-61], were also over-represented among the genes in our dataset. Of particular relevance to behavioral deficits in ASD, genes related to cognition, learning, repetitive behaviors (for example, circling), memory, and spatial learning are also associated with these potential targets of RORA.

Among the selected targets of RORA investigated in this study, we confirmed *ITPR1* and *CYP19A1* as transcriptional targets in human neuronal cells by both ChIP-quantitative PCR and functional knockdown of RORA with shRNA. As mentioned earlier, both genes had been implicated as targets of RORA by earlier studies with Rora-deficient mice [29] as well as in human breast cancer [36]. Our current study, however, also reveals four novel transcriptional targets of RORA, thus expanding the repertoire of genes and pathways that may be impacted by RORA deficiency in humans. These novel target genes are *A2BP1 (RBFOX1)*, *HSD17B10*, *NLGN1*, and *NTRK2*. Furthermore, our pilot study examining the expression of these six transcriptional targets of RORA in postmortem brain tissues from individuals with ASD and that of age-matched controls shows an average reduction of all targets in the autism samples, although the differences between combined cases and combined controls were not statistically significant, all exhibiting *P* >0.05. Surprisingly, the least significant differences in average expression levels were observed for *RORA* and *CYP19A1*. Inasmuch as we had previously detected significant differences (*P* <0.05) between the protein expression levels of both RORA and CYP19A1 in the frontal cortex of 22 cases versus 12 controls by confocal immunofluorescence analyses [12], we suggest that the lack of statistical significance for reduced expression of these two transcripts as well as the other targets in this study is probably due to the limitation in sample size (*n* = 3 for each group) coupled with the natural variability of gene expression among the individuals. Nevertheless, the data from this limited sampling of cases and controls suggest a trend towards reduced expression of transcriptional targets in autism brain tissues exhibiting reduced RORA. As discussed below, an independent review of the literature revealed that these six RORA targets were reliably associated with autism.

### Relevance of *A2BP1, CYP19A1, HSD17B10, ITPR1, NLGN1,* and *NTRK2* to the pathobiology of autism

A2BP1 (ataxin 2-binding protein 1), also known as RBFOX1, is an RNA-binding protein that regulates neuron-specific alternative splicing [62]. Several studies, including genetic and gene expression analyses, have established the link between this gene and autism. Using fluorescent *in situ* hybridization and quantitative PCR analyses, Martin and colleagues found a cryptic deletion of the *A2BP1* gene in a female with autism, epilepsy, and global development delay [33]. Reduction of the *A2BP1* transcript level was also observed in the lymphocytes of this individual, suggesting that the deletion causes aberrant expression of this gene in this autism case. By genotyping 27 SNPs across this gene in 206 parent–child trios, they identified two regions exhibiting a nominal association with autism. Moreover, a recent noise-reduction genome-wide association study (GWAS) of two autism GWAS datasets (with 597 and 696 families) from the Autism Genetic Resource Exchange revealed 1,535 significant linkage disequilibrium blocks overlapping 431 genes [63]. Interestingly, regions in the *A2BP1* gene were identified among the most significant linkage disequilibrium blocks (*P* = 3.6×10^-5^). Furthermore, Voineagu and colleagues also conducted transcriptomic analysis of postmortem frontal and temporal cortex tissues from 19 individuals with autism and 17 controls using microarrays [64]. They found that *A2BP1* expression was significantly reduced in both frontal and temporal cortex tissues from individuals with autism relative to controls. Using high-throughput RNA sequencing and semi-quantitative RT-PCR analyses, they also demonstrated that splicing of A2BP1-dependent alternative exons in the brain of individuals with autism was dysregulated [64], suggesting that aberrant expression of *A2BP1* results in dysregulation of alternative splicing in autism. Findings from several copy number variation (CNV) studies [65-70] and linkage studies [71-75] have also reported that *A2BP1* is associated with autism. Aside from its association with ASD, recent studies on the physiological function of A2BP1/RBFOX1 demonstrate its involvement in synaptic transmission and neuronal excitation [76] as well as its role in the regulation of transcriptional networks involved in neuronal development [77].

*CYP19A1* encodes aromatase, a key enzyme that converts androstenedione to estrone and testosterone to estradiol. Although its neuronal function is most often associated with the regulation of reproductive behaviors through the sex hormones, more recent studies have revealed unexpected functions of aromatase in the brain, including neurogenesis, neuronal differentiation, synaptic activity and plasticity, and protection against oxidative stress [50,78-82]. Another interesting observation is the colocalization of aromatase with oxytocin in several regions of the rat brain, including the periventricular nucleus of the hypothalamus and the zona incerta, which suggests a role for this gene in oxytocinergic neurons in the limbic system [83], which may have implications for social cognition. *CYP19A1* has also been associated with autism by genetic studies. Allen-Brady and colleagues conducted genome-wide screening of 70 families (192 individuals with autism and 461 unaffected relatives) and identified three regions shown to be highly significant in the linkage analysis [84]. Interestingly, one of the three regions is 15q21.1-15q22.2 (heterogeneity logarithm (base 10) of the odds = 5.31) where the *CYP19A1* gene is located. Moreover, Chakrabarti and colleagues studied SNPs in 68 candidate genes for autism to identify common genetic variations associated with autistic traits and Asperger syndrome, a high-functioning subgroup of autism. A case–control association analysis of individuals with Asperger syndrome (*n* = 174) revealed regions in 14 genes, including *CYP19A1*, that showed a nominally significant association with Asperger syndrome [85].

*HSD17B10* encodes 3-hydroxyacyl-CoA dehydrogenase type-2, a mitochondrial enzyme involved in mitochondrial integrity and oxidation of fatty acids and steroids. As mentioned earlier, like CYP19A1, HSD17B10 is involved in the conversion of androgens to estradiol via androstenedione and estrone intermediates. A reduction in HSD17B10 would thus be expected to lead to increased androgen and reduced estrogen, either of which may have serious consequences for brain development [86]. Indeed, reduced expression of *HSD17B10* causes X-linked mental retardation, choreoathetosis, language impairment, and abnormal behavior [87-89]. These associated disorders are particularly interesting since we have identified RORA deficiency (specifically, reduced expression) in the phenotypic subtype of ASD associated with severe language impairment [13]. Using chromosome microarray analysis, Edens and colleagues reported duplications involving Xp11.22-p11.23, a region where *HSD17B10* gene is located, in two females with autism and epilepsy [90].

*ITPR1* encodes inositol 1,4,5-triphosphate receptor type 1, a ligand-gated ion channel that is activated by cytosolic calcium and inositol triphosphate, and that is involved in synaptogenesis and formation of dendritic contacts [91]. Computational modeling also suggests that the biochemical and biophysical properties of ITPR1 coupled with its high density and lower sensitivity to IP_3_ in cerebellar Purkinje cells are critical to its postulated role in long-term depression [92]. With respect to ASD, transcriptomic analysis of frontal and temporal cortex tissues from 16 individuals with autism and 16 controls revealed down-regulation of *ITPR1*[64]. Bremer and colleagues used high-resolution whole genome array-based comparative genomic hybridization to screen 223 individuals with autism to identify gene dose alterations associated with autism susceptibility. They found regions in chromosome 3p25.3, where *ITPR1* is located, significantly enriched in individuals with autism [93]. Another CNV study of 1,124 families, each of which included a single proband, his/her unaffected parents and, in most cases, an unaffected sibling, revealed as many as 234 CNV regions in several genes, including *ITPR1*, to be associated with autism [69]. Moreover, Levy and colleagues studied genomic copy-number variation in a large cohort of families with a single affected child and at least one unaffected sibling. Among significant regions identified to be *de novo* CNVs were regions containing the *ITPR1* gene [94].

*NLGN1* encodes neuroligin-1, a neuronal cell surface adhesion molecule that interacts with neurexin molecules. NLGN1 is involved in formation and remodeling of synapses in the central nervous system [95]. In addition to synaptogenesis, a recent study has also demonstrated a role for NLGN1, acting together with NRXN1β, in neuritogenesis and the formation of neuronal circuits upon activation of the fibroblast growth factor receptor-1 [96]. In the context of ASD, Glessner and colleagues conducted a whole-genome CNV analysis of 859 individuals with autism and 1,409 typically developing children of European ancestry who were genotyped with ~550,000 SNP markers to comprehensively identify CNVs conferring susceptibility to autism [97]. Positive findings were evaluated in an independent cohort of 1,336 individuals with autism and 1,110 controls of European ancestry. Interestingly, a CNV region in the *NLGN1* gene was significantly enriched in individuals with autism. Moreover, a family-based association analysis of 100 families with autism found a modest significant association at a region in *NLGN1* gene [98]. A high-density SNP, genome-wide linkage study also showed that the chromosomal region 3q26.31-q27.3 was significantly associated with autism [99]. Deletion of this gene was also reported to cause impaired spatial memory and increased repetitive behavior [100].

*NTRK2* encodes neurotrophic tyrosine kinase receptor type 2 (also known as TRKB) that is a catalytic receptor for several neurotrophins, including BDNF, NT4, and NT3. NTRK2 is involved in axon guidance signaling as well as synapse formation and plasticity [101-104]. Its role in ASD is implicated by genetic association analysis of 146 children with autism and 50 typically developing controls which identified six SNPs and multiple haplotypes in the *NTRK2* gene [105]. This study also showed that BDNF levels were significantly increased in children with autism relative to that in controls, supporting a role of NTRK2/BDNF signaling as a susceptibility factor for the disorder.

### Study limitations and future directions

Because little is known with regard to transcriptional targets of RORA, particularly in human neuronal cells, this ChIP-on-chip analysis was undertaken as a discovery tool to screen for potential transcriptional targets at the genome-wide level. As a result, we used a less stringent *P*-value of 0.05 as a filter to select probes enriched for RORA binding without applying correction for multiple testing (which is not part of the standard Partek analytical workflow for tiling arrays). However, all of the regions identified using *P* ≤0.05 are retained in the dataset even after imposing FDR ≤7%. Thus, although the list of 2,544 genes that are identified as putative targets of RORA should be interpreted with caution pending independent validation, we suggest that this list may be used as a basis for hypothesis generation with regard to the genes, pathways and functions that may be impacted by RORA deficiency. Another caveat is that the list of RORA targets does not replicate all of the several targets identified in the mouse cerebellum by ChIP-quantitative PCR analyses [29] other than *ITPR1*. We do not know the precise reason for this difference, but it is possible that the target selection may be a function of the specific tissues and cell types as well as species, since the mouse targets were identified in primary cerebellar cells, and the human targets were identified in the SH-SY5Y human neuroblastoma cell line. Indeed, co-regulator availability is also tissue-specific, and transcriptional regulation by RORA requires association with gene-specific and tissue-specific co-regulator proteins, thus adding another layer of complexity to target selection.

Another potential complication is the possibility that target selection may be directed by circadian rhythm, as RORA is a regulator of the circadian cycle. Thus, it would be interesting to conduct ChIP-on-chip analysis on cells synchronized according to stage in the circadian cycle. Finally, as noted earlier, although the average expression levels of the RORA targets were reduced in the frontal cortex of individuals with ASD relative to that of controls as expected, the differences were not statistically significant. Notably, the protein expression levels of the genes exhibiting the least significant expression differences (that is, highest *P* values) in this study, RORA and CYP19A1, were found to be significantly different in our previous study in which 12 autism tissue samples were compared with 22 controls [12], thus emphasizing the need for an expanded investigation of RORA targets involving additional brain tissues from individuals with ASD and controls, including samples from different brain regions.

**Figure 4:**
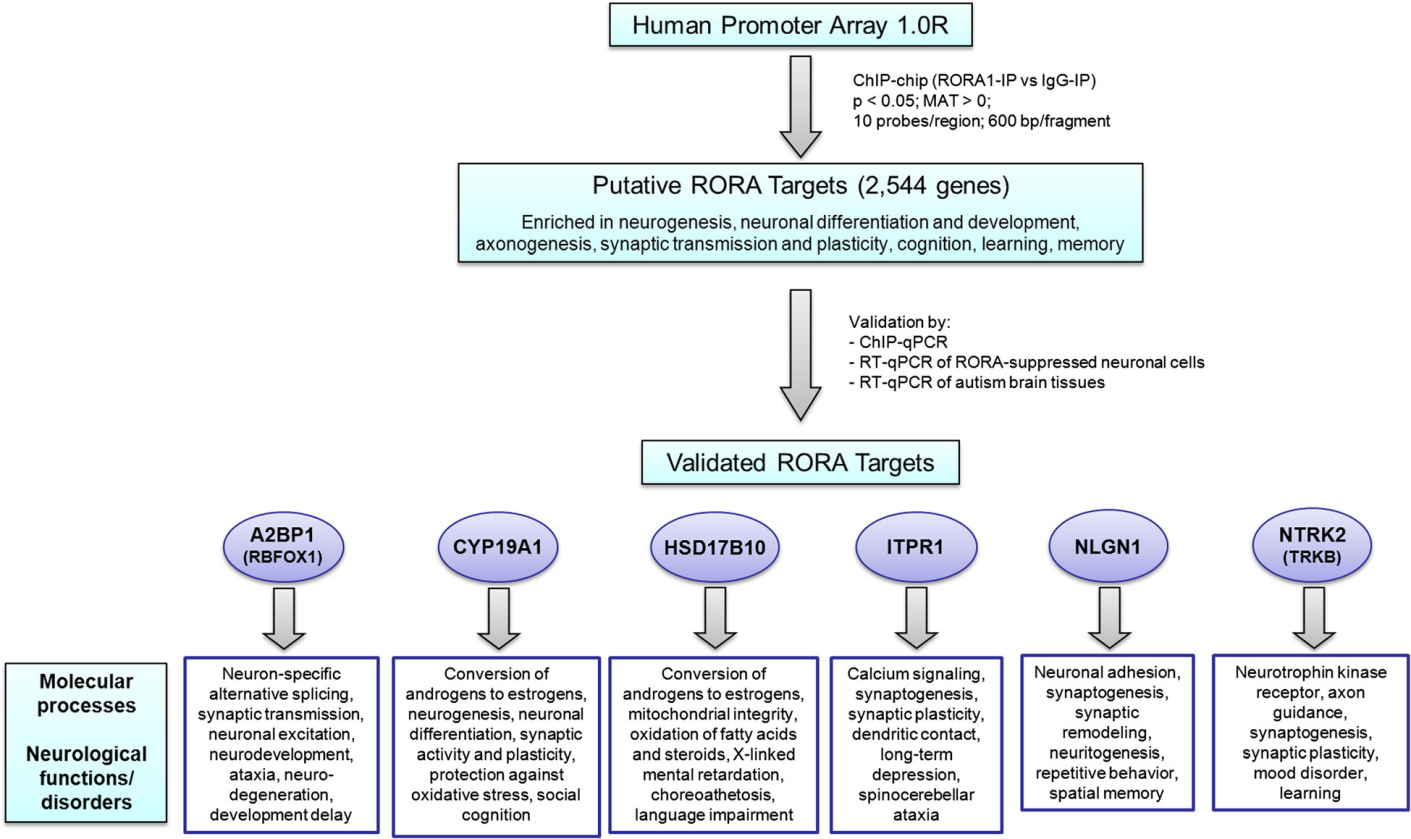
**Possible downstream consequences of deregulation of the six confirmed transcriptional targets of RORA.** This figure summarizes findings from this study. ChIP, chromatin immunoprecipitation; qPCR, quantitative PCR.

In summary, using a high-throughput genome-wide method for identifying potential transcriptional targets of RORA followed by independent validation of six targets with functional relevance to ASD, we have demonstrated that RORA deficiency can lead to a cascade of transcriptional deregulation that impacts a number of genes known to be associated with ASD. Figure 4 summarizes the principal findings from this study.

## Conclusions

Using ChIP-on-chip analyses, we identify at the genome-wide level over 2,500 potential transcriptional targets of RORA, a nuclear hormone receptor, and confirm six of these targets (four of them novel with respect to regulation by RORA) in human neuronal cells by ChIP-quantitative PCR and functional analyses. Moreover, we demonstrate that the transcript levels of these target genes are reduced in the postmortem frontal cortex of autistic males who exhibit reduced RORA expression relative to that of control males, further implicating RORA as an upstream regulator of these genes in human brain. The significance of these findings is the likelihood that any mechanism that contributes to reduced *RORA* expression, including elevated levels of male hormones [12] and epigenetic modifications that may be the result of as yet unknown environmental factors [11], may lead to increased risk for autism based on the aberrant transcription of downstream targets of RORA, which we show are significantly enriched for autism candidate genes based on hypergeometric distribution analyses of our dataset in comparison with established databases of autism genes.

## Abbreviations

A2BP1: Ataxin 2 binding protein 1; Acsl4: Acyl-CoA synthetase long-chain family member 4; Akt2: V-akt murine thymoma viral oncogene homolog 2; ASD: Autism spectrum disorder; BA: Brodmann area; BDNF: brain-derived neurotrophic factor; bp: base pairs; Calb1: Calbindin 1, 28kDa; Cals: Callose synthase; Cd36: Trombospondin receptor; ChIP: chromatin immunoprecipitation; ChIP-on-chip: chromatin immunoprecipitation followed by microarray analysis; Cidea: Cell death-inducing DFFA-like effector a; Cidec: Cell death-inducing DFFA-like effector c; CNV: Copy number variant(s); CYP19A1: Cytochrome P450, family 19, subfamily A, polypeptide 1; FDR: False discovery rate; GABA: Gamma aminobutyric acid; Grm1: Glutamate receptor, metabotropic 1; Hif1a: Hypoxia inducible factor 1, alpha subunit; HSD17B10: Hydroxysteroid (17-β) dehydrogenase 10; IPA: Ingenuity Pathway Analysis; ITPR1: Inositol 1,4,5-trisphosphate receptor, type 1; LCL: Lymphoblastoid cell line; LXRa: Liver X receptor alpha; MEM: modified Eagle’s medium; Mogat1: Monoacylglycerol O-acyltransferase 1; NLGN1: Neuroligin 1; NLGN3: Neuroligin 3; NRXN1: Neurexin 1; NT3: Neurotransmitter transporter 3; NT4: Neurotransmitter transporter 4; NTRK2: Neurotrophic tyrosine kinase, receptor, type 2; PBS: phosphate-buffered saline; Pcp2: Purkinje cell protein 2; Pcp4: Purkinje cell protein 4; PCR: polymerase chain reaction; RBFOX1: RNA-binding protein, fox-1 homolog; RELN: Reelin; RORA: Retinoic acid receptor-related orphan receptor alpha; RORA1: Retinoic acid receptor-related orphan receptor alpha, isoform 1; RT: Reverse transcription; Shh: Sonic hedgehog; shRNA: Short hairpin RNA; SH-SY5Y: Human neuroblastoma cell line; Slc1a6: Solute carrier protein 1, member 6; SNP: Single-nucleotide polymorphism; Srebp-1c: Sterol regulatory element-binding protein 1c

## Competing interests

The authors declare that they have no competing interests.

## Authors' contributions

TS and VWH conceived of the study, contributed to the study design, performed the data analyses, and prepared the manuscript. TS conducted the experiments. All authors read and approved the final manuscript.

## Supplementary Material

Additional file 1 A table presenting a list of frozen human brain tissue samples from the Autism Tissue Program. Click here for file

Additional file 2 A table presenting a list of antibodies and shRNAs. Click here for file

Additional file 3 A table presenting a list of primers for ChIP-quantitative PCR, and quantitative RT- PCR. Click here for file

Additional file 4 A table presenting a list of potential transcriptional targets of RORA identified by ChIP-on-chip analysis. Click here for file

Additional file 5 A table presenting annotation clusters 2 to 5 resulting from gene ontology analysis of the ChIP-on-chip gene dataset using DAVID Bioinformatics Resources 6.7. Click here for file

Additional file 6 A table presenting a complete list of genes associated with neurological functions, disorders, and behaviors identified by Ingenuity Pathway Analysis as significantly over-represented in ChIP-on-chip dataset of RORA-binding target promoters. Click here for file

## References

[B1] American Psychiatric Association Task Force on DSM-IV: Diagnostic and statistical manual of mental disorders: DSM-IV-TR 2000 4 Washington, DC: American Psychiatric Association

[B2] Moldin SO Rubenstein JLR Understanding autism: from basic neuroscience to treatment 2006 CRC/Taylor & Francis: Boca Raton

[B3] Volkmar F Chawarska K Klin A Autism in infancy and early childhood Annu Rev Psychol 2005 56 315 336 10.1146/annurev.psych.56.091103.070159 15709938

[B4] Volkmar FR Klin A Siegel B Szatmari P Lord C Campbell M Freeman BJ Cicchetti DV Rutter M Kline W Field trial for autistic disorder in DSM-IV Am J Psychiatry 1994 151 1361 1367 8067493 10.1176/ajp.151.9.1361

[B5] Baio J Prevalence of Autism Spectrum Disorders - Autism and Developmental Disabilities Monitoring Network, 14 Sites, United States, 2008 Centers for Disease Control and Prevention Morbidity and Mortality Weekly Report 2012 61 3 22456193

[B6] Kim YS Leventhal BL Koh Y Fombonne E Laska E Lim E Cheon K Kim S Kim Y Lee H Song D Grinker RR Prevalence of autism spectrum disorders in a total population sample Am J Psychiatry 2011 168 904 912 10.1176/appi.ajp.2011.10101532 21558103

[B7] Mattila M Kielinen M Linna S Jussila K Ebeling H Bloigu R Joseph RM Moilanen I Autism spectrum disorders according to DSM-IV-TR and comparison with DSM-5 draft criteria: an epidemiological study J Am Acad Child Adolesc Psychiatry 2011 50 583 592 10.1016/j.jaac.2011.04.001 21621142

[B8] Baron-Cohen S Knickmeyer RC Belmonte MK Sex differences in the brain: implications for explaining autism Science 2005 310 819 823 10.1126/science.1115455 16272115

[B9] Auyeung B Baron-Cohen S Ashwin E Knickmeyer R Taylor K Hackett G Fetal testosterone and autistic traits Br J Psychol 2009 100 Pt 1 1 22 18547459 10.1348/000712608X311731

[B10] Auyeung B Taylor K Hackett G Baron-Cohen S Foetal testosterone and autistic traits in 18 to 24-month-old children Mol Autism 2010 1 11 10.1186/2040-2392-1-11 20678186 PMC2916006

[B11] Nguyen A Rauch TA Pfeifer GP Hu VW Global methylation profiling of lymphoblastoid cell lines reveals epigenetic contributions to autism spectrum disorders and a novel autism candidate gene, RORA, whose protein product is reduced in autistic brain FASEB J 2010 24 3036 3051 10.1096/fj.10-154484 20375269 PMC2909294

[B12] Sarachana T Xu M Wu RC Hu VW Sex hormones in autism: androgens and estrogens differentially and reciprocally regulate RORA, a novel candidate gene for autism PLoS One 2011 6 e17116 10.1371/journal.pone.0017116 21359227 PMC3040206

[B13] Hu VW Sarachana T Kim KS Nguyen A Kulkarni S Steinberg ME Luu T Lai Y Lee NH Gene expression profiling differentiates autism case-controls and phenotypic variants of autism spectrum disorders: evidence for circadian rhythm dysfunction in severe autism Autism Res 2009 2 78 97 10.1002/aur.73 19418574 PMC2737477

[B14] Boukhtouche F Janmaat S Vodjdani G Gautheron V Mallet J Dusart I Mariani J Retinoid-related orphan receptor alpha controls the early steps of Purkinje cell dendritic differentiation J Neurosci 2006 26 1531 1538 10.1523/JNEUROSCI.4636-05.2006 16452676 PMC6675487

[B15] Hadj-Sahraoui N Frederic F Zanjani H Delhaye-Bouchaud N Herrup K Mariani J Progressive atrophy of cerebellar Purkinje cell dendrites during aging of the heterozygous staggerer mouse (Rora(+/sg)) Brain Res Dev Brain Res 2001 126 201 209 10.1016/S0165-3806(01)00095-5 11248354

[B16] Gold DA Gent PM Hamilton BA ROR alpha in genetic control of cerebellum development: 50 staggering years Brain Res 2007 1140 19 25 16427031 10.1016/j.brainres.2005.11.080

[B17] Harding HP Atkins GB Jaffe AB Seo WJ Lazar MA Transcriptional activation and repression by RORalpha, an orphan nuclear receptor required for cerebellar development Mol Endocrinol 1997 11 1737 1746 10.1210/me.11.11.1737 9328355

[B18] Boukhtouche F Vodjdani G Jarvis CI Bakouche J Staels B Mallet J Mariani J Lemaigre-Dubreuil Y Brugg B Human retinoic acid receptor-related orphan receptor alpha1 overexpression protects neurones against oxidative stress-induced apoptosis J Neurochem 2006 96 1778 1789 10.1111/j.1471-4159.2006.03708.x 16539693

[B19] Delerive P Monte D Dubois G Trottein F Fruchart-Najib J Mariani J Fruchart JC Staels B The orphan nuclear receptor ROR alpha is a negative regulator of the inflammatory response EMBO Rep 2001 2 42 48 10.1093/embo-reports/kve007 11252722 PMC1083804

[B20] Sato TK Panda S Miraglia LJ Reyes TM Rudic RD McNamara P Naik KA FitzGerald GA Kay SA Hogenesch JB A functional genomics strategy reveals Rora as a component of the mammalian circadian clock Neuron 2004 43 527 537 10.1016/j.neuron.2004.07.018 15312651

[B21] Fatemi SH Aldinger KA Ashwood P Bauman ML Blaha CD Blatt GJ Chauhan A Chauhan V Dager SR Dickson PE Estes AM Goldowitz D Heck DH Kemper TL King BH Martin LA Millen KJ Mittleman G Mosconi MW Persico AM Sweeney JA Webb SJ Welsh JP Consensus paper: pathological role of the cerebellum in autism Cerebellum 2012 11 777 807 10.1007/s12311-012-0355-9 22370873 PMC3677555

[B22] Bourgeron T The possible interplay of synaptic and clock genes in autism spectrum disorders Cold Spring Harb Symp Quant Biol 2007 72 645 654 10.1101/sqb.2007.72.020 18419324

[B23] Wimpory D Nicholas B Nash S Social timing, clock genes and autism: a new hypothesis J Intellect Disabil Res 2002 46 Pt 4 352 358 12000587 10.1046/j.1365-2788.2002.00423.x

[B24] Melke J Goubran Botros H Chaste P Betancur C Nygren G Anckarsater H Rastam M Stahlberg O Gillberg IC Delorme R Chabane N Mouren-Simeoni MC Fauchereau F Durand CM Chevalier F Drouot X Collet C Launay JM Leboyer M Gillberg C Bourgeron T Abnormal melatonin synthesis in autism spectrum disorders Mol Psychiatry 2008 13 90 98 10.1038/sj.mp.4002016 17505466 PMC2199264

[B25] Lalonde R Strazielle C Discrimination learning in Rora(sg) and Grid2(ho) mutant mice Neurobiol Learn Mem 2008 90 2 472 474 10.1016/j.nlm.2008.05.004 18583162

[B26] Goodall G Gheusi G Abnormal patterns of maze patrolling in the mutant mouse staggerer Behav Neural Biol 1987 47 307 320 10.1016/S0163-1047(87)90422-5 3606530

[B27] Lalonde R Botez MI Boivin D Object exploration in staggerer mutant mice Physiol Behav 1987 41 115 117 10.1016/0031-9384(87)90139-9 3685159

[B28] Lalonde R Exploration and spatial learning in staggerer mutant mice J Neurogenet 1987 4 285 291 3440888

[B29] Gold DA Baek SH Schork NJ Rose DW Larsen DD Sachs BD Rosenfeld MG Hamilton BA RORalpha coordinates reciprocal signaling in cerebellar development through sonic hedgehog and calcium-dependent pathways Neuron 2003 40 1119 1131 10.1016/S0896-6273(03)00769-4 14687547 PMC2717708

[B30] Raichur S Fitzsimmons RL Myers SA Pearen MA Lau P Eriksson N Wang SM Muscat GE Identification and validation of the pathways and functions regulated by the orphan nuclear receptor, ROR alpha1, in skeletal muscle Nucleic Acids Res 2010 38 4296 4312 10.1093/nar/gkq180 20338882 PMC2910057

[B31] Kang HS Okamoto K Takeda Y Beak JY Gerrish K Bortner CD DeGraff LM Wada T Xie W Jetten AM Transcriptional profiling reveals a role for RORalpha in regulating gene expression in obesity-associated inflammation and hepatic steatosis Physiol Genomics 2011 43 818 828 10.1152/physiolgenomics.00206.2010 21540300 PMC3132837

[B32] Johnson WE Li W Meyer CA Gottardo R Carroll JS Brown M Liu XS Model-based analysis of tiling-arrays for ChIP-chip Proc Natl Acad Sci U S A 2006 103 12457 12462 10.1073/pnas.0601180103 16895995 PMC1567901

[B33] Martin CL Duvall JA Ilkin Y Simon JS Arreaza MG Wilkes K Alvarez-Retuerto A Whichello A Powell CM Rao K Cook E Geschwind DH Cytogenetic and molecular characterization of A2BP1/FOX1 as a candidate gene for autism Am J Med Genet B Neuropsychiatr Genet 2007 144B 869 876 10.1002/ajmg.b.30530 17503474

[B34] Basu SN Kollu R Banerjee-Basu S AutDB: A gene reference resource for autism research Nucleic Acids Res 2009 37 Suppl 1 D832 D836 19015121 10.1093/nar/gkn835 PMC2686502

[B35] Xu L Li J Huang Y Zhao M Tang X Wei L AutismKB: an evidence-based knowledgebase of autism genetics Nucleic Acids Res 2012 40 D1 D1016 D1022 10.1093/nar/gkr1145 22139918 PMC3245106

[B36] Odawara H Iwasaki T Horiguchi J Rokutanda N Hirooka K Miyazaki W Koibuchi Y Shimokawa N Iino Y Takeyoshi I Koibuchi N Activation of aromatase expression by retinoic acid receptor-related orphan receptor (ROR) alpha in breast cancer cells: identification of a novel ROR response element J Biol Chem 2009 284 17711 17719 10.1074/jbc.M109.009241 19439415 PMC2719410

[B37] Farre D Roset R Huerta M Adsuara JE Rosello L Alba MM Messeguer X Identification of patterns in biological sequences at the ALGGEN server: PROMO and MALGEN Nucleic Acids Res 2003 31 3651 3653 10.1093/nar/gkg605 12824386 PMC169011

[B38] Messeguer X Escudero R Farre D Nunez O Martinez J Alba MM PROMO: detection of known transcription regulatory elements using species-tailored searches Bioinformatics 2002 18 333 334 10.1093/bioinformatics/18.2.333 11847087

[B39] Bryne JC Valen E Tang MH Marstrand T Winther O da Piedade I Krogh A Lenhard B Sandelin A JASPAR, the open access database of transcription factor-binding profiles: new content and tools in the 2008 update Nucleic Acids Res 2008 36 D102 D106 10.1093/nar/gkn449 18006571 PMC2238834

[B40] Rozen S Skaletsky H Primer3 on the WWW for general users and for biologist programmers Methods Mol Biol 2000 132 365 386 10547847 10.1385/1-59259-192-2:365

[B41] Hu VW Nguyen A Kim KS Steinberg ME Sarachana T Scully MA Soldin SJ Luu T Lee NH Gene expression profiling of lymphoblasts from autistic and nonaffected sib pairs: altered pathways in neuronal development and steroid biosynthesis PLoS One 2009 4 e5775 10.1371/journal.pone.0005775 19492049 PMC2685981

[B42] Johnson NL Kotz S Kemp AW Univariate Discrete Distributions 1992 New York: Wiley

[B43] Huang DW Sherman BT Lempicki RA Systematic and integrative analysis of large gene lists using DAVID bioinformatics resources Nat Protoc 2009 4 44 57 19131956 10.1038/nprot.2008.211

[B44] Huang DW Sherman BT Lempicki RA Bioinformatics enrichment tools: paths toward the comprehensive functional analysis of large gene lists Nucleic Acids Res 2009 37 1 13 10.1093/nar/gkn923 19033363 PMC2615629

[B45] Amaral DG Schumann CM Nordahl CW Neuroanatomy of autism Trends Neurosci 2008 31 137 145 10.1016/j.tins.2007.12.005 18258309

[B46] Bourgeron T A synaptic trek to autism Curr Opin Neurobiol 2009 19 231 234 10.1016/j.conb.2009.06.003 19545994

[B47] Carper RA Courchesne E Localized enlargement of the frontal cortex in early autism Biol Psychiatry 2005 57 126 133 10.1016/j.biopsych.2004.11.005 15652870

[B48] Courchesne E Pierce K Why the frontal cortex in autism might be talking only to itself: local over-connectivity but long-distance disconnection Curr Opin Neurobiol 2005 15 225 230 10.1016/j.conb.2005.03.001 15831407

[B49] Kelleher RJ Bear MF 3rd The autistic neuron: troubled translation? Cell 2008 135 401 406 10.1016/j.cell.2008.10.017 18984149

[B50] Wegiel J Kuchna I Nowicki K Imaki H Wegiel J Marchi E Ma SY Chauhan A Chauhan V Bobrowicz TW de Leon M Louis LA Cohen IL London E Brown WT Wisniewski T The neuropathology of autism: defects of neurogenesis and neuronal migration, and dysplastic changes Acta Neuropathol 2010 119 755 770 10.1007/s00401-010-0655-4 20198484 PMC2869041

[B51] Konstantareas MM Hewitt T Autistic disorder and schizophrenia: diagnostic overlaps J Autism Dev Disord 2001 31 19 28 10.1023/A:1005605528309 11439750

[B52] Volkmar FR Cohen DJ Comorbid association of autism and schizophrenia Am J Psychiatry 1991 148 1705 1707 1957933 10.1176/ajp.148.12.1705

[B53] Ghaziuddin M Butler E Clumsiness in autism and Asperger syndrome: a further report J Intellect Disabil Res 1998 42 43 48 10.1046/j.1365-2788.1998.00065.x 9534114

[B54] Jones V Prior M Motor imitation abilities and neurological signs in autistic children J Autism Dev Disord 1985 15 37 46 10.1007/BF01837897 3980428

[B55] Kielinen M Rantala H Timonen E Linna SL Moilanen I Associated medical disorders and disabilities in children with autistic disorder: a population-based study Autism 2004 8 49 60 10.1177/1362361304040638 15070547

[B56] Minshew NJ Sung K Jones BL Furman JM Underdevelopment of the postural control system in autism Neurology 2004 63 2056 2061 10.1212/01.WNL.0000145771.98657.62 15596750

[B57] Vilensky JA Damasio AR Maurer RG Gait disturbances in patients with autistic behavior: a preliminary study Arch Neurol 1981 38 646 649 10.1001/archneur.1981.00510100074013 7295109

[B58] Hartley-McAndrew M Weinstock A Autism Spectrum Disorder: Correlation between aberrant behaviors, EEG abnormalities and seizures Neurology Int 2010 2 42 47 10.4081/ni.2010.e10 PMC3093215 21577334

[B59] Robinson SJ Childhood epilepsy and autism spectrum disorders: psychiatric problems, phenotypic expression, and anticonvulsants Neuropsychol Rev 2012 22 271 279 10.1007/s11065-012-9212-3 22875726

[B60] Lesca G Rudolf G Labalme A Hirsch E Arzimanoglou A Genton P Motte J de Saint Martin A Valenti M Boulay C De Bellescize J Kéo-Kosal P Boutry-Kryza N Edery P Sanlaville D Szepetowski P Epileptic encephalopathies of the Landau-Kleffner and continuous spike and waves during slow-wave sleep types: genomic dissection makes the link with autism Epilepsia 2012 53 1526 1538 10.1111/j.1528-1167.2012.03559.x 22738016

[B61] Berg AT Plioplys S Epilepsy and autism: is there a special relationship? Epilepsy Behav 2012 23 193 198 10.1016/j.yebeh.2012.01.015 22381386 PMC3307824

[B62] Zhou H Baraniak AP Lou H Role for Fox-1/Fox-2 in mediating the neuronal pathway of calcitonin/calcitonin gene-related peptide alternative RNA processing Mol Cell Biol 2007 27 830 841 10.1128/MCB.01015-06 17101796 PMC1800674

[B63] Hussman JP Chung RH Griswold AJ Jaworski JM Salyakina D Ma D Konidari I Whitehead PL Vance JM Martin ER Cuccaro ML Gilbert JR Haines JL Pericak-Vance MA A noise-reduction GWAS analysis implicates altered regulation of neurite outgrowth and guidance in autism Mol Autism 2011 2 1 10.1186/2040-2392-2-1 21247446 PMC3035032

[B64] Voineagu I Wang X Johnston P Lowe JK Tian Y Horvath S Mill J Cantor RM Blencowe BJ Geschwind DH Transcriptomic analysis of autistic brain reveals convergent molecular pathology Nature 2011 474 380 384 10.1038/nature10110 21614001 PMC3607626

[B65] Autism Genome Project C Szatmari P Paterson AD Zwaigenbaum L Roberts W Brian J Liu XQ Vincent JB Skaug JL Thompson AP Senman L Feuk L Qian C Bryson SE Jones MB Marshall CR Scherer SW Vieland VJ Bartlett C Mangin LV Goedken R Segre A Pericak-Vance MA Cuccaro ML Gilbert JR Wright HH Abramson RK Betancur C Bourgeron T Gillberg C Mapping autism risk loci using genetic linkage and chromosomal rearrangements Nat Genet 2007 39 319 328 10.1038/ng1985 17322880 PMC4867008

[B66] Bucan M Abrahams BS Wang K Glessner JT Herman EI Sonnenblick LI Alvarez Retuerto AI Imielinski M Hadley D Bradfield JP Kim C Gidaya NB Lindquist I Hutman T Sigman M Kustanovich V Lajonchere CM Singleton A Kim J Wassink TH McMahon WM Owley T Sweeney JA Coon H Nurnberger JI Li M Cantor RM Minutesshew NJ Sutcliffe JS Cook EH Genome-wide analyses of exonic copy number variants in a family-based study point to novel autism susceptibility genes PLoS Genet 2009 5 e1000536 10.1371/journal.pgen.1000536 19557195 PMC2695001

[B67] Gai X Xie HM Perin JC Takahashi N Murphy K Wenocur AS D'arcy M O'Hara RJ Goldmuntz E Grice DE Shaikh TH Hakonarson H Buxbaum JD Elia J White PS Rare structural variation of synapse and neurotransmission genes in autism Mol Psychiatry 2012 17 402 411 10.1038/mp.2011.10 21358714 PMC3314176

[B68] Pinto D Pagnamenta AT Klei L Anney R Merico D Regan R Conroy J Magalhaes TR Correia C Abrahams BS Almeida J Bacchelli E Bader GD Bailey AJ Baird G Battaglia A Berney T Bolshakova N Bolte S Bolton PF Bourgeron T Brennan S Brian J Bryson SE Carson AR Casallo G Casey J Chung BH Cochrane L Corsello C Functional impact of global rare copy number variation in autism spectrum disorders Nature 2010 466 368 372 10.1038/nature09146 20531469 PMC3021798

[B69] Sanders SJ Ercan-Sencicek AG Hus V Luo R Murtha MT Moreno-De-Luca D Chu SH Moreau MP Gupta AR Thomson SA Mason CE Bilguvar K Celestino-Soper PB Choi M Crawford EL Davis L Wright NR Dhodapkar RM DiCola M DiLullo NM Fernandez TV Fielding-Singh V Fishman DO Frahm S Garagaloyan R Goh GS Kammela S Klei L Lowe JK Lund SC Multiple recurrent de novo CNVs, including duplications of the 7q11.23 Williams syndrome region, are strongly associated with autism Neuron 2011 70 863 885 10.1016/j.neuron.2011.05.002 21658581 PMC3939065

[B70] Sebat J Lakshmi B Malhotra D Troge J Lese-Martin C Walsh T Yamrom B Yoon S Krasnitz A Kendall J Leotta A Pai D Zhang R Lee YH Hicks J Spence SJ Lee AT Puura K Lehtimaki T Ledbetter D Gregersen PK Bregman J Sutcliffe JS Jobanputra V Chung W Warburton D King MC Skuse D Geschwind DH Gilliam TC Strong association of de novo copy number mutations with autism Science 2007 316 445 449 10.1126/science.1138659 17363630 PMC2993504

[B71] Buxbaum JD Silverman J Keddache M Smith CJ Hollander E Ramoz N Reichert JG Linkage analysis for autism in a subset families with obsessive-compulsive behaviors: evidence for an autism susceptibility gene on chromosome 1 and further support for susceptibility genes on chromosome 6 and 19 Mol Psychiatry 2004 9 144 150 10.1038/sj.mp.4001465 14699429

[B72] International Molecular Genetic Study of Autism Consortium A full genome screen for autism with evidence for linkage to a region on chromosome 7q Hum Mol Genet 1998 7 571 578 9546821 10.1093/hmg/7.3.571

[B73] International Molecular Genetic Study of Autism Consortium A genomewide screen for autism: strong evidence for linkage to chromosomes 2q, 7q, and 16p Am J Hum Genet 2001 69 570 581 11481586 10.1086/323264 PMC1235486

[B74] Lauritsen MB Als TD Dahl HA Flint TJ Wang AG Vang M Kruse TA Ewald H Mors O A genome-wide search for alleles and haplotypes associated with autism and related pervasive developmental disorders on the Faroe Islands Mol Psychiatry 2006 11 37 46 10.1038/sj.mp.4001754 16205737

[B75] McCauley JL Li C Jiang L Olson LM Crockett G Gainer K Folstein SE Haines JL Sutcliffe JS Genome-wide and Ordered-Subset linkage analyses provide support for autism loci on 17q and 19p with evidence of phenotypic and interlocus genetic correlates BMC Med Genet 2005 6 1 15647115 10.1186/1471-2350-6-1 PMC546213

[B76] Gehman LT Stoilov P Maguire J Damianov A Lin C Shiue L Ares M Mody I Black DL The splicing regulator Rbfox1 (A2BP1) controls neuronal excitation in the mammalian brain Nat Genet 2011 43 706 711 10.1038/ng.841 21623373 PMC3125461

[B77] Fogel BL Wexler E Wahnich A Friedrich T Vijayendran C Gao F Parikshak N Konopka G Geschwind DH RBFOX1 regulates both splicing and transcriptional networks in human neuronal development Hum Mol Genet 2012 21 4171 4186 10.1093/hmg/dds240 22730494 PMC3441119

[B78] Garcia-Segura LM Aromatase in the brain: not just for reproduction anymore J Neuroendocrinol 2008 20 705 712 10.1111/j.1365-2826.2008.01713.x 18601693

[B79] Garcia-Segura LM Veiga S Sierra A Melcangi RC Azcoitia I Aromatase: a neuroprotective enzyme Prog Neurobiol 2003 71 31 41 10.1016/j.pneurobio.2003.09.005 14611865

[B80] Rune GM Frotscher M Neurosteroid synthesis in the hippocampus: Role in synaptic plasticity Neuroscience 2005 136 833 842 10.1016/j.neuroscience.2005.03.056 16344155

[B81] Chauhan A Chauhan V Oxidative stress in autism Pathophysiology 2006 13 171 181 10.1016/j.pathophys.2006.05.007 16766163

[B82] McGinnis WR Oxidative stress in autism Altern Ther Health Med 2004 10 22 36 15624347

[B83] El-Emam Dief A Caldwell JD Jirikowski GF Colocalization of P450 Aromatase and Oxytocin Immunostaining in the Rat Hypothalamus Horm and Metab Res 2012 45 273 276 23225240 10.1055/s-0032-1327680

[B84] Allen-Brady K Robison R Cannon D Varvil T Villalobos M Pingree C Leppert MF Miller J McMahon WM Coon H Genome-wide linkage in Utah autism pedigrees Mol Psychiatry 2010 15 1006 1015 10.1038/mp.2009.42 19455147 PMC4023913

[B85] Chakrabarti B Dudbridge F Kent L Wheelwright S Hill-Cawthorne G Allison C Banerjee-Basu S Baron-Cohen S Genes related to sex steroids, neural growth, and social-emotional behavior are associated with autistic traits, empathy, and Asperger syndrome Autism Res 2009 2 157 177 10.1002/aur.80 19598235

[B86] Yang S He X Miller D Hydroxysteroid (17β) dehydrogenase X in human health and disease Mol Cell Endocrinol 2011 343 1 6 10.1016/j.mce.2011.06.011 21708223

[B87] Lenski C Kooy RF Reyniers E Loessner D Wanders RJ Winnepenninckx B Hellebrand H Engert S Schwartz CE Meindl A Ramser J The reduced expression of the HADH2 protein causes X-linked mental retardation, choreoathetosis, and abnormal behavior Am J Hum Genet 2007 80 372 377 10.1086/511527 17236142 PMC1785340

[B88] Ofman R Ruiter JP Feenstra M Duran M Poll-The BT Zschocke J Ensenauer R Lehnert W Sass JO Sperl W Wanders RJ 2-Methyl-3-hydroxybutyryl-CoA dehydrogenase deficiency is caused by mutations in the HADH2 gene Am J Hum Genet 2003 72 1300 1307 10.1086/375116 12696021 PMC1180283

[B89] Reyniers E Van Bogaert P Peeters N Vits L Pauly F Fransen E Van Regemorter N Kooy RF A new neurological syndrome with mental retardation, choreoathetosis, and abnormal behavior maps to chromosome Xp11 Am J Hum Genet 1999 65 1406 1412 10.1086/302638 10521307 PMC1288294

[B90] Edens AC Lyons MJ Duron RM Dupont BR Holden KR Autism in two females with duplications involving Xp11.22-p11.23 Dev Med Child Neurol 2011 53 463 466 10.1111/j.1469-8749.2010.03909.x 21418194

[B91] Banerjee S Hasan G The InsP3 receptor: its role in neuronal physiology and neurodegeneration Bioessays 2005 27 1035 1047 10.1002/bies.20298 16163728

[B92] Hernjak N Slepchenko BM Fernald K Fink CC Fortin D Moraru II Watras J Loew LM Modeling and analysis of calcium signaling events leading to long-term depression in cerebellar Purkinje cells Biophys J 2005 89 3790 3806 10.1529/biophysj.105.065771 16169982 PMC1366947

[B93] Bremer A Giacobini M Eriksson M Gustavsson P Nordin V Fernell E Gillberg C Nordgren A Uppstromer A Anderlid BM Nordenskjold M Schoumans J Copy number variation characteristics in subpopulations of patients with autism spectrum disorders Am J Med Genet B Neuropsychiatr Genet 2011 156 115 124 10.1002/ajmg.b.31142 21302340

[B94] Levy D Ronemus M Yamrom B Lee YH Leotta A Kendall J Marks S Lakshmi B Pai D Ye K Buja A Krieger A Yoon S Troge J Rodgers L Iossifov I Wigler M Rare de novo and transmitted copy-number variation in autistic spectrum disorders Neuron 2011 70 886 897 10.1016/j.neuron.2011.05.015 21658582

[B95] Scheiffele P Fan J Choih J Fetter R Serafini T Neuroligin expressed in nonneuronal cells triggers presynaptic development in contacting axons Cell 2000 101 657 669 10.1016/S0092-8674(00)80877-6 10892652

[B96] Gjørlund MD Nielsen J Pankratova S Li S Korshunova I Bock E Berezin V Neuroligin-1 induces neurite outgrowth through interaction with neurexin-1β and activation of fibroblast growth factor receptor-1 FASEB Journal 2012 26 4174 4186 10.1096/fj.11-202242 22750515

[B97] Glessner JT Wang K Cai G Korvatska O Kim CE Wood S Zhang H Estes A Brune CW Bradfield JP Imielinski M Frackelton EC Reichert J Crawford EL Munson J Sleiman PM Chiavacci R Annaiah K Thomas K Hou C Glaberson W Flory J Otieno F Garris M Soorya L Klei L Piven J Meyer KJ Anagnostou E Sakurai T Autism genome-wide copy number variation reveals ubiquitin and neuronal genes Nature 2009 459 569 573 10.1038/nature07953 19404257 PMC2925224

[B98] Ylisaukko-oja T Rehnstrom K Auranen M Vanhala R Alen R Kempas E Ellonen P Turunen JA Makkonen I Riikonen R Nieminen-von Wendt T von Wendt L Peltonen L Jarvela I Analysis of four neuroligin genes as candidates for autism Eur J Hum Genet 2005 13 1285 1292 10.1038/sj.ejhg.5201474 16077734

[B99] Allen-Brady K Miller J Matsunami N Stevens J Block H Farley M Krasny L Pingree C Lainhart J Leppert M McMahon WM Coon H A high-density SNP genome-wide linkage scan in a large autism extended pedigree Mol Psychiatry 2009 14 590 600 10.1038/mp.2008.14 18283277

[B100] Blundell J Blaiss CA Etherton MR Espinosa F Tabuchi K Walz C Bolliger MF Sudhof TC Powell CM Neuroligin-1 deletion results in impaired spatial memory and increased repetitive behavior J Neurosci 2010 30 2115 2129 10.1523/JNEUROSCI.4517-09.2010 20147539 PMC2824441

[B101] Shen K Cowan CW Guidance molecules in synapse formation and plasticity Cold Spring Harbor Pers Biol 2010 2 a001842 10.1101/cshperspect.a001842 PMC2845208 20452946

[B102] Luikart BW Nef S Virmani T Lush ME Liu Y Kavalali ET Parada LF TrkB has a cell-autonomous role in the establishment of hippocampal schaffer collateral synapses J Neurosci 2005 25 3774 3786 10.1523/JNEUROSCI.0041-05.2005 15829629 PMC6724922

[B103] Lu Y Christian K Lu B BDNF: a key regulator for protein synthesis-dependent LTP and long-term memory? Neurobiol Learn Mem 2008 89 312 323 10.1016/j.nlm.2007.08.018 17942328 PMC2387254

[B104] Huang EJ Reichardt LF Trk receptors: Roles in neuronal signal transduction Annu Rev Biochem 2003 72 609 642 10.1146/annurev.biochem.72.121801.161629 12676795

[B105] Correia CT Coutinho AM Sequeira AF Sousa IG Lourenco Venda L Almeida JP Abreu RL Lobo C Miguel TS Conroy J Cochrane L Gallagher L Gill M Ennis S Oliveira GG Vicente AM Increased BDNF levels and NTRK2 gene association suggest a disruption of BDNF/TrkB signaling in autism Genes Brain Behav 2010 9 841 848 10.1111/j.1601-183X.2010.00627.x 20662941

